# Multidimensional and Multifunctional Laser‐Induced Graphene (LIG) for Point‐of‐Care and Wearable Biosensing, Theranostics, and Bioactive Interfaces Toward Personalized Healthcare and Regenerative Medicine

**DOI:** 10.1002/advs.75238

**Published:** 2026-04-13

**Authors:** Li Zhang, Yurou Yuan, Xiaohong Ding, Wing Cheung Mak

**Affiliations:** ^1^ Department of Biomedical Engineering The Chinese University of Hong Kong Hong Kong China

**Keywords:** bioactive interfaces, biosensors, laser‐induced graphene (LIG), personalized healthcare, regenerative medicine, theranostics

## Abstract

Multidimensional laser‐induced graphene (LIG) has emerged as an attractive signal transduction and bioactive material, functioning both as high‐performance biosensors and multifunctional biomedical platforms. Its unique porous, graphene‐like morphology offers multiple advantageous features, including high conductivity, tunable surface energy, high active surface area, catalytic activity, biocompatibility, and mechanical flexibility, along with facile fabrication through a chemical‐free, mask‐free, and programmable laser writing process. Yet, the exploration of its dimensional versatility, spanning from 0D to 3D architectures, facilitates multifunctional biomedical applications for personalized healthcare and regenerative medicine remain challenging. This review summarizes recent advances in LIG‐based platforms for personalized healthcare and regenerative medicine, beginning with LIG‐based transducers for various biosensing modalities, including physiochemical, point‐of‐care, and wearable biosensing. We further examine LIG‐based platforms serving as bioactive interfaces for advanced multifunctional biomedical applications, including anti‐microbial strategies, smart drug delivery, closed‐loop theranostic platforms, lab‐on‐a‐chip systems, and regenerative medicine applications such as tissue engineering and cell scaffolding. Emerging directions, including LIG‐optics, LIG‐based organ‐on‐a‐chip platforms, smart 4D LIG material, and AI‐powered LIG systems, are also discussed. By bridging LIG's multidimensional architectures and multifunctional capabilities, this review highlights the potential of LIG materials to advance the development of future personalized healthcare and regenerative medical systems.

## Graphical abstract

Multidimensional laser‐induced graphene (LIG) spanning from 0D to 3D architectures is comprehensively reviewed for multifunctional biomedical platforms, including biosensing, theranostics, and bioactive interface applications, which highlights its potentials for point‐of‐care diagnostics, wearable health monitoring, smart drug delivery, and tissue engineering, toward personalized healthcare and regenerative medicine.

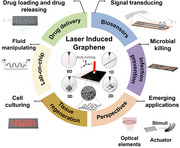

## 1 Introduction

The healthcare field is undergoing a profound transformation from centralized clinical settings toward decentralized approaches that emphasize point‐of‐care diagnostics and home‐based monitoring [1]. This systemic transition, driven by the vision of personalized healthcare with tailored medical support for individual patients, demands innovative technologies capable of providing accessible, portable, and user‐friendly health monitoring and management [2]. Portable and wearable biosensor technologies have emerged as transformative biomedical devices in response to these demands, effectively bridging the gap between laboratory diagnostics and practical everyday health monitoring [3]. Portable point‐of‐care biosensor devices enable rapid, on‐site diagnostics through discrete measurements for conditions such as blood glucose monitoring [4], cardiac biomarker detection [5, 6], and infectious disease screening [7], while wearable biosensors integrate into patients' daily routines to provide continuous, minimally invasive monitoring of vital signs [8], activity levels, and biochemical markers [9]. Recent advances have further extended beyond biosensing into theranostic platforms that integrate real‐time diagnostic capabilities with therapeutic interventions, creating closed‐loop systems for personalized healthcare management [10].

The biosensor typically comprises three essential components, including biorecognition elements, transducers, and signal processors, where the transducer serves as the critical interface that converts biological interactions into measurable electrical, optical, or mechanical signals [11]. Contemporary transducer design and fabrication have evolved to incorporate multidimensionality as an architectural strategy, which can modulate functions across diverse biomedical applications. For instance, the point or single‐line fabrication creates 0D quantum dots and nanoparticles [12], or 1D wires and fibers [13, 14, 15]; the planar patterning generates 2D electrode patterns on planar substrates [16], while the multi‐layer assembly and layer‐by‐layer additive manufacturing enable 3D hierarchical transducer structures [17, 18, 19, 20]. This dimensional control allows functional modulation of transducers tailored to the specific portable and wearable biosensing platforms, for example, 0D/1D transducers suit compact devices requiring the localized detection [13], 2D transducers enable lateral flow devices [21] and skin‐conformal patches [22], and 3D transducers can be integrated with microfluidics for complex sample handling and manipulation [23].

Among diverse fabrication methods to construct multidimensional transducers, laser‐induced graphene (LIG) based on laser direct writing [24] has emerged as a particularly promising technology to fabricate 0–3D architecture transducers [25]. LIG employs direct laser writing to convert carbon‐rich precursors into highly conductive graphene/graphite material via photothermal or photochemical processes. The versatility of LIG stems from its multidimensional fabrication capabilities: laser spot irradiation or single‐line scanning produces 0D LIG quantum dots [12] or 1D LIG fibers [13, 14, 15], respectively; laser planar scanning fabricates 2D patterned LIG electrodes on various synthetic and natural‐derived substrates (e.g. PI film and cellulose paper) [26, 27, 28, 29] and multilayer fabrication enables 3D hierarchical LIG transducer architectures [17, 18, 19, 20]. This dimensional tunability, together with the porous morphology, high active surface area, tunable surface energy, superior catalytic activity, and the favorable biocompatibility of LIG, collectively support the fabrication of multidimensional high‐performance LIG transducers‐based biosensor devices [30, 31, 32]. Importantly, the capabilities of LIG extend well beyond biosensing into multifunctional biomedical applications essential for comprehensive personalized healthcare and regenerative medicine. The porous architecture and electrical conductivity that enable high electrochemical biosensing performance also provide mechanisms for establishing bioactive interfaces including antimicrobial protection [33], programmable drug delivery [34] and microfluidic manipulation [35], as well as for building regenerative medicine platforms such as tissue scaffold engineering and cell‐interfacing substrates [36], alongside thermal actuation for nucleic acid amplification [37]. This multifunctionality enables the integration of diagnostic and therapeutic capabilities within unified theranostic platforms, representing a critical advancement toward closed‐loop healthcare systems capable of real‐time health monitoring and personalized therapeutics.

**FIGURE 1:**
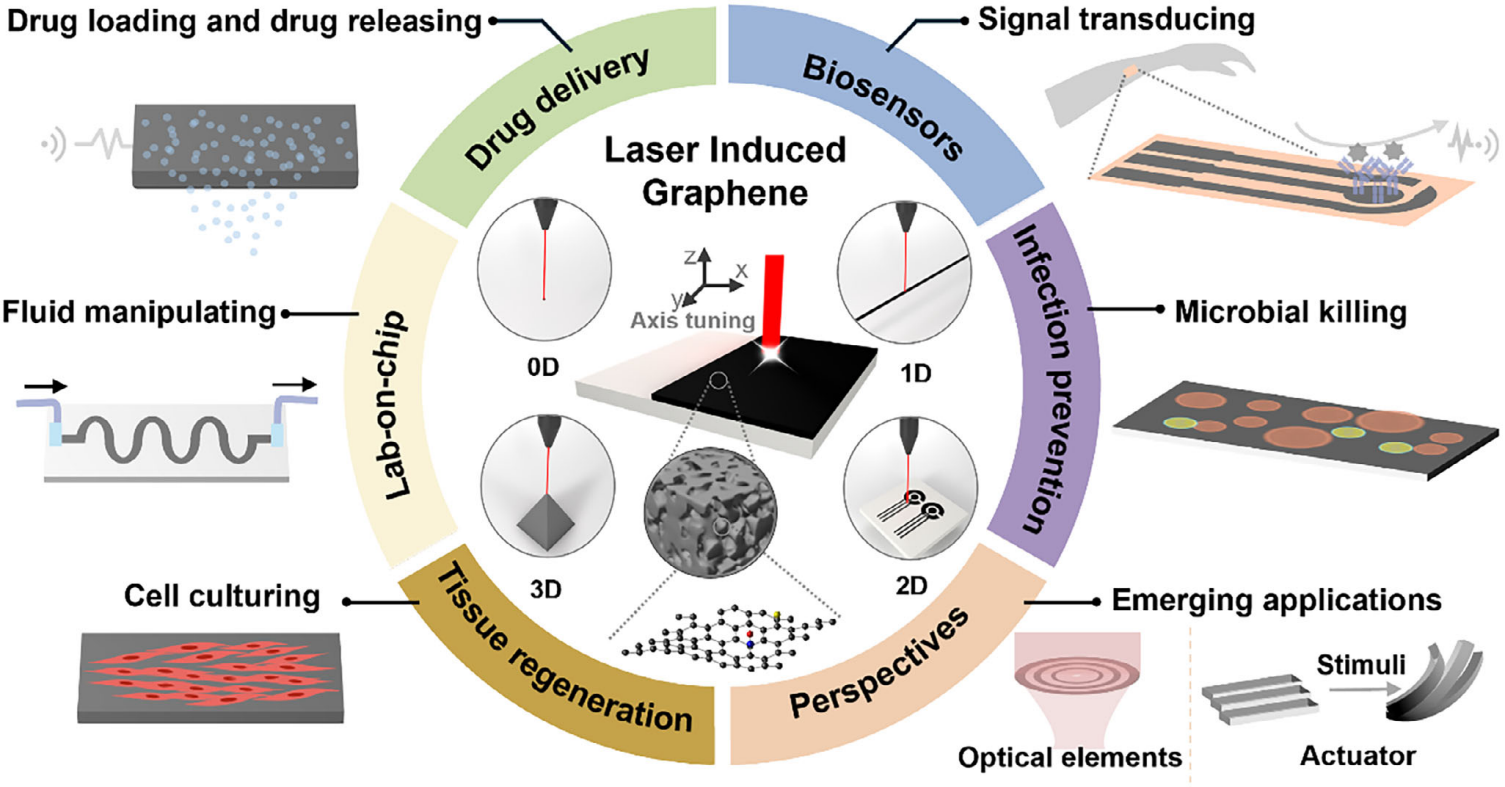
Schematic of multidimensional LIG for multifunctional applications. Some figure elements were created by BioGDP.com.

As illustrated in Figure 1, this review provides a comprehensive overview of multidimensional and multifunctional LIG for point‐of‐care and wearable biosensing, theranostic applications, and bioactive interfaces toward personalized healthcare and regenerative medicine. We begin by elucidating the fundamental principles of LIG formation and systematically examining multidimensional LIG spanning 0–3D architectures. We then explore LIG transducers for biosensing toward personalized healthcare, covering physical and physiological monitoring, including strain, electrophysiology, temperature, and humidity sensing, followed by electrochemical detection of biochemical markers in point‐of‐care, wearable, and implantable platforms. Moving beyond biosensing, we examine LIG‐based platforms serving as bioactive interfaces for multifunctional biomedical applications, including anti‐microbial interfaces, smart drug delivery, and theranostic platforms, lab‐on‐a‐chip systems, alongside nucleic acid amplification. We further explore the role of LIG in regenerative medicine, including tissue engineering scaffolds and cell culture substrates for stem cell applications and tissue regeneration. Finally, we discuss future perspectives on LIG‐optics, LIG‐based organ‐on‐a‐chip platforms, the emerging 4D LIG concepts with time‐dependent and stimuli‐responsive functionality, and AI‐powered LIG‐based biosensors. Through this systematic examination from fundamental principles to frontier applications, we envision intelligent and autonomous LIG‐based platforms incorporated into biomedical systems for personalized medicine and regenerative medicine.

## 2 Fundamental of Multidimensional LIG

### 2.1 Fundamentals of LIG

Carbon‐based materials, including fullerenes, carbon nanotubes (CNTs), graphene, and their derivatives, have profoundly transformed biomedical research and applications over the past several decades [38, 39]. Among these materials, graphene and graphene‐like carbon materials have emerged as particularly attractive candidates for biomedical applications owing to their outstanding physicochemical properties, including high electrical conductivity (10^6^ S/cm) [40], theoretical specific surface area up to ∼2630 m^2^ g^−1^ (for single‐layer graphene) [41], and favorable biocompatibility [42]. This growing interest has driven the development of numerous synthesis approaches, including mechanical exfoliation, reduction of graphite oxide, epitaxial growth, chemical vapor deposition (CVD) [43], and LIG [41].

Among these techniques, LIG fabrication has emerged as a particularly compelling method that directly converts carbon‐rich precursors into graphene/graphene‐like materials through photothermal laser‐induced graphitization [41]. During the laser‐induced fabrication, intense energy is applied at the precursor materials, which induces atomic vibrations that generate localized temperatures reaching around 3000 K to break covalent bonds (e.g., C–O, C = O, N–C) and induce the rearrangement of carbon atoms, converting the sp^3^‐hybridized carbon structure into the sp^2^‐hybridized graphene/graphitic structure. Simultaneously, gases are rapidly released from this process, resulting in the formation of the LIG with porous and interconnected graphene/graphite nanostructures having a high specific surface areas reaching 340 m^2^ g^−1^ [44, 45, 46].

This chemical‐free and mask‐free approach offers high versatility, demonstrating the compatibility with various commercial laser systems (e.g., CO_2_, visible light, UV lasers) and diverse carbon‐rich precursors such as polyimide (PI), polyether ether ketone (PEEK), and lignin [47]. Therefore, this versatility endows LIG with a suite of taliorable properties such as tunable electrical conductivity, porous nanostructures, surface energy, and wettability. The electrical conductivity of LIG fabricated on PI substrates under optimized CO_2_ laser conditions (25 µm laser spot size, 50 µm line spacing, and 15 mm s^−1^ scan speed) exhibits approximately 3290 S m^−1^ [48]. This high electrical conductivity enables LIG to function as a competent signal transducers in both physical and electrochemical biosensors, with high electrochemical activity achieving low charge–transfer resistance (∼120 Ω) that supports high‐performance physiological sensing [49]. The nanostructures of LIG can also be regulated by controlling laser parameters, such as the transition of the LIG structure from sheet‐like porous nanostructures to vertically aligned nanofibers reaching heights up to 1 mm by increasing the laser fluence beyond 40 J/cm^2^ [50]. This porous nanostructure provides abundant anchoring and active sites for the functionalization of conductive polymers, immobilization of biological components, and deposition of functional nanomaterials that integrate with LIG as heterostructured transducers [51, 52, 53]. In addition, the surface energy and wetting behavior of LIG can be modulated through the fabrication atmosphere and laser parameters. The LIG fabrication in the oxygen atmosphere yields a super‐hydrophilic LIG surface with water contact angles approaching 0°, whereas processing in argon or hydrogen produces a superhydrophobic LIG surface with contact angles exceeding 150° [54, 55]. This tunable surface energy and wettability enable the fabrication of lab‐on‐chip biomedical devices with fluidic manipulation abilities [35, 56, 57]. Moreover, the tunable properties of LIG is highly dependent on the choice of precursor substrates and laser processing parameters, including laser type, fluence, power, scanning speed, spot size, and number of laser scans, which has been discussed in other review article [31]. Some examples of LIG fabricated under different precursor substrates, laser types, and processing parameters, and their resulting transducer architectures and device applications, are summarized in Table 1.

**TABLE 1:** Versatility of multidimensional LIG for multifunctional biomedical application.

LIG composite	Dimension	Laser type	Parameters	Post treatments	Application	Refs.
LIG/PDMS	0D	522 nm femtosecond laser	Laser power, laser scanning speed	N/A	Optoelectrical devices	[12]
LIG/PI monofilament	1D	CO_2_ laser	Laser power, pulse resolution	N/A	Physical sensor (airflow breathing)	[13]
LIG/PI film	1D	UV laser	Laser power, laser scanning speed, laser spot size	N/A	Helical millirobots	[63]
	2D	UV laser	Laser fluence	LiCl deposited	Physical sensor (humidity)	[64]
	2D	CO_2_ laser	N/A	Au deposited, DNA probes immobilized	Electrochemical sensor (miRNA)	[65]
	2D	Femtosecond fiber laser	Laser scanning speeds	N/A	Anti‐icing/deicing	[66]
	2D	CO_2_ laser	Laser power	N/A	Pressure sensor (gait recognition)	[67]
LIG/Fe_3_O_4_ coated PI film	2D	UV laser	N/A	N/A	Drug delivery	[68]
LIG/paraffin coated paper	2D	CO_2_ laser	Laser power, laser scanning speed, number of laser scans	PtNPs deposited	Physical sensor (strain), capacitor, electrochemical sensor (H_2_O_2_)	[69]
LIG/graphene oxide coated bacterial cellulose	2D	405 nm laser	N/A	PANI, PPY deposited	Physical (strain) and electrochemical sensor (pH)	[70]
LIG/cellulose	2D	CO_2_ laser	Laser power, laser scanning speed, defocus distance	Silver epoxy deposited	Electrochemical sensor (P. aeruginosa)	[71]
LIG/lignin	2D	CO_2_ laser	Laser power, laser scanning speed	PB deposited, and LOx immobilized	Electrochemical sensor (H_2_O_2_ and lactate)	[53]
LIG/MnO coated wood	2D	Yb‐doped fiber laser	Laser fluences, laser scanning speed	N/A	Supercapacitor	[72]
LIG/PI	3D	CO_2_ laser	N/A	PVA/H_2_SO_4_ stacked	Supercapacitor	[73]
	3D	CO_2_ laser	N/A	PVA stacked	Supercapacitor	[20]
	3D	CO_2_ laser	N/A	PPY, Cipro, PANI, PEDOT, and PB deposited, Uricase immobilized	Drug delivery, electrochemical sensor (pH and UA)	[34]
	3D	CO_2_ laser and 1.06 µm fiber laser	N/A	N/A	Physical sensor (pulse)	[19]
	3D	CO_2_ laser	Laser power	N/A	Triboelectric nanogenerator	[74]
	3D	CO_2_ laser	Laser power, laser scanning speed	Silicone rubber stacked	Physical sensor (motion)	[75]
LIG/lignocellulose	3D	CO_2_ laser	Laser power, laser scanning speed, number of laser scans	N/A	Seawater desalination, electrodes	[76]

Abbreviations: N/A: not applicable; UA: Uric acid; PtNPs: Platinum nanoparticles; PPY: Polypyrrole; PANI: Polyaniline; PB: Prussian blue; LOx: Lactate oxidase; PEDOT: Poly(3,4‐ethylenedioxythiophene);

Additionally, LIG has demonstrated a favorable biocompatibility assessed using zebrafish models, demonstrating its suitability for establishing bioactive interfaces that support cell/tissue‐interfacing devices and regenerative medicine platforms, including tissue scaffolds and implantable therapeutic systems [58]. LIG‐based patches and electrodes have demonstrated stable long‐term performance with minimal inflammatory response, which further supports its biomedical application in implantable devices [59]. LIG also exhibits optical properties because of the porous and interconnected graphene/graphite nanostructure that demonstrates broadband optical absorption spanning from UV to infrared wavelengths, with absorption coefficients reaching approximately 97%–99% across the 280–2500 nm spectral range [60]. This high optical absorptivity enables photothermal conversion, where the absorbed light energy can be transformed into localized heat with biomedical applications in photothermal [61] and electro‐photo‐thermal antimicrobial devices [62].

### 2.2 Multidimensional LIG Architecture

LIG exhibits unique architecture versatility that stems from the controllable laser fabrication process, enabling the production of LIG with customized geometries and dimensions that span from 0D and 1D into 2D and 3D architectures. The localized point [12] or line laser irradiation [13, 14, 15] produces 0D and 1D LIG structures respectively, while scanning on planar substrates generates the 2D patterned LIG [16]. 3D LIG architecture can be fabricated either through additive manufacturing that creates monolithic LIG objects or through the sequential stacking of multiple 2D LIG layers [17, 18, 19, 20].

**FIGURE 2:**
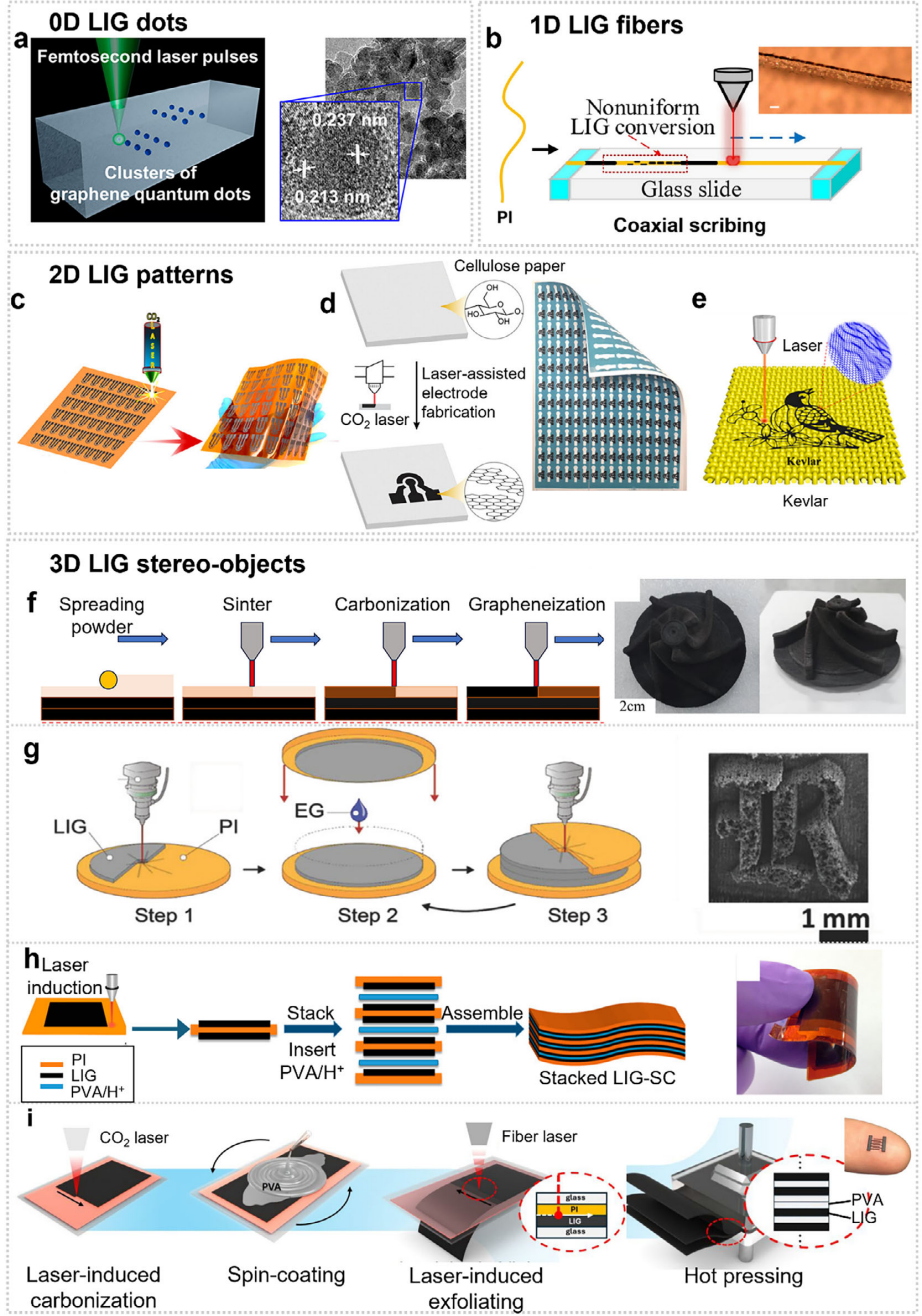
Representatives of multidimensional LIG architecture and fabrication process. (a) 0D LIG dots fabricated using femtosecond laser pulses. Reproduced with permission [12]. Copyright 2021, American Chemical Society. (b) 1D LIG fibers fabricated via a vertically oriented laser‐sweeping strategy. Reproduced with permission [13]. Copyright 2020, Elsevier. (c) 2D LIG electrode patterns fabricated on polyimide films. Reproduced with permission [78]. Copyright 2019, American Chemical Society. (d) 2D LIG electrode patterns fabricated on cellulose papers. Reproduced with permission [21]. Copyright 2023, Wiley‐VCH. (e) 2D LIG patterns fabricated on white Kevlar textiles. Reproduced with permission [79]. Copyright 2020, American Chemical Society. (f) A 3D LIG architecture fabricated via additive manufacturing, including layer‐by‐layer sintering, carbonization, and graphitization. Reproduced with permission [76]. Copyright 2024, Elsevier. (g) 3D LIG stereo‐objects fabricated by laminated object manufacturing. Reproduced with permission [19]. Copyright 2018, Wiley‐VCH. (h) A 3D LIG‐based supercapacitor prepared via vertically stacking a 2D film. Reproduced with permission [73]. Copyright 2015, American Chemical Society. (i) Stacked 3D LIG‐based electrodes prepared through the stacked laminate fabrication process. Reproduced with permission [20]. Copyright 2025, American Chemical Society.

Specifically, the localized point fabrication by direct femtosecond laser pulse writing in liquid media such as polydimethylsiloxane (PDMS) produces luminescent graphene quantum dots (GQDs) as 0D LIG dots (Figure 2a) [12]. These 0D LIG dots, endowed with unique quantum confinement effects, offer strong, tunable fluorescence suitable for bioimaging and photoluminescent detection of biomolecules (e.g., DNA, proteins, metabolites) in point‐of‐care platforms [77]. 1D laser‐sweeping fabrication strategies can produce 1D LIG fibers with concentric graphene layers forming cylindrical shells around a polyimide core and fiber diameters of 10–20 µm and aspect ratios exceeding 1000:1. This 1D LIG material architecture provides high mechanical flexibility, directional conductivity, and embeddability, making 1D LIG fibers particularly attractive for integration into smart textiles and wearable electronic systems (Figure 2b) [13].

The programmable laser writing process of LIG enables the facile patterning of customized 2D LIG electrodes on a wide variety of planar substrates, including polymer films [78], cellulose papers [21], and textiles [79], allowing transducer designs to be readily tailored for specific biomedical applications (Figure 2c–e). Among polymer film substrates, commercial PI films were first demonstrated as effective polymer film substrates for 2D LIG fabrication (Figure 2c) [78], and subsequent investigations have expanded the material library to include polycarbonate (PC) [80], polyetherimide (PEI) [28], polyether ether ketone (PEEK) [81] and phenolic resin (PR) [82]. Beyond polymer films, 2D LIGs can be patterned on cellulose papers to create transducers that leverage the inherent capillary transport abilities of the paper matrix, enabling the development of microfluidic paper‐based analytical devices (µPADs) tailored for point‐of‐care diagnostics such as the quantitative detection of HPV16 (Figure 2d) [21]. Additionally, versatile 2D LIG transducers have been directly patterned onto Kevlar textiles to develop intelligent protective clothing tailored to specific wearable scenarios, while preserving the intrinsic flexibility and breathability of the textile substrate (Figure 2e) [79]. The combination of versatile substrate compatibility, customizable electrode patterning, and thin flexible form factors makes 2D LIGs particularly attractive for portable and wearable biosensing applications where conformability, lightweight design, and application‐specific configurations are essential.

Through additive manufacturing, 3D LIG architectures can be fabricated using an approach analogous to selective laser sintering (SLS) 3D printing [18]. This technology utilizes multistep, layer‐by‐layer laser processing to selectively sinter the desired regions and subsequently graphitize them, enabling the production of multifunctional 3D LIG objects for sensing and actuation applications (Figure 2f) [76]. Inspired by stereolithography (SLA) 3D printing, the production of 3D LIG objects from liquid precursors has also been demonstrated, yielding interconnected graphene/graphite networks with surface areas exceeding 39.3 cm^2^ g^−1^ and specific electrical conductivity of approximately 162.8 S cm^−2^ g^−1^ [17]. In addition, the laminated object manufacturing (LOM) technique employs stacking, laser conversion, fusing, and milling of PI films to produce 3D LIG architectures with controllable porosity and conductivity (Figure 2g) [19]. Similarly, 3D LIG supercapacitors can also be fabricated by stacking and sandwiching approaches (Figure 2h) [73]. Hot‐pressing has also been combined with stacking to fabricate thickness‐tunable, flexible 3D LIG objects that exhibit high electrical conductivity (∼1100 S m^−1^), high mechanical robustness, and rapid electrothermal heating ability (17°C s^−1^) (Figure 2i) [20].

### 2.3 Multidimensional LIG Transducers for Biosensing

Transducers are critical components in physical [83], point‐of‐care [4], portable, and wearable biosensing devices [9] that enable the signal conversion by translating the interaction between biological elements (bioreceptors) and analytes (target substances being detected) into a measurable signal. Multidimensional LIG transducers are attractive for biosensing applications owing to their ability to produce high‐performance conductive elements with desired dimensions and geometries.

#### 2.3.1 Physical Biosensing

Physical biosensing relies on transduction mechanisms that convert mechanical, thermal, or environmental stimuli into quantifiable electrical signals. The principal mechanisms underpinning LIG‐based physical biosensors include piezoresistive, resistive, capacitive, and triboelectric transduction [5, 6]. In piezoresistive sensing, mechanical deformation of the conductive LIG network alters inter‐graphene contact resistance and charge transport pathways, producing measurable resistance changes proportional to the applied strain or pressure. In resistive sensing, variations in environmental conditions such as temperature or humidity modulate the bulk electrical resistance of the LIG network through carrier scattering or molecular adsorption mechanisms. Capacitive sensing exploits changes in the dielectric properties or electrode separation induced by mechanical deformation or surface interactions. These complementary transduction mechanisms collectively underpin the diverse physical biosensing capabilities of LIG transducers, spanning strain and pressure detection, electrophysiological biosensing, and temperature and humidity monitoring, each of which is examined in detail in the following subsections.

##### 2.3.1.1 Strain and Pressure Biosensing

**FIGURE 3:**
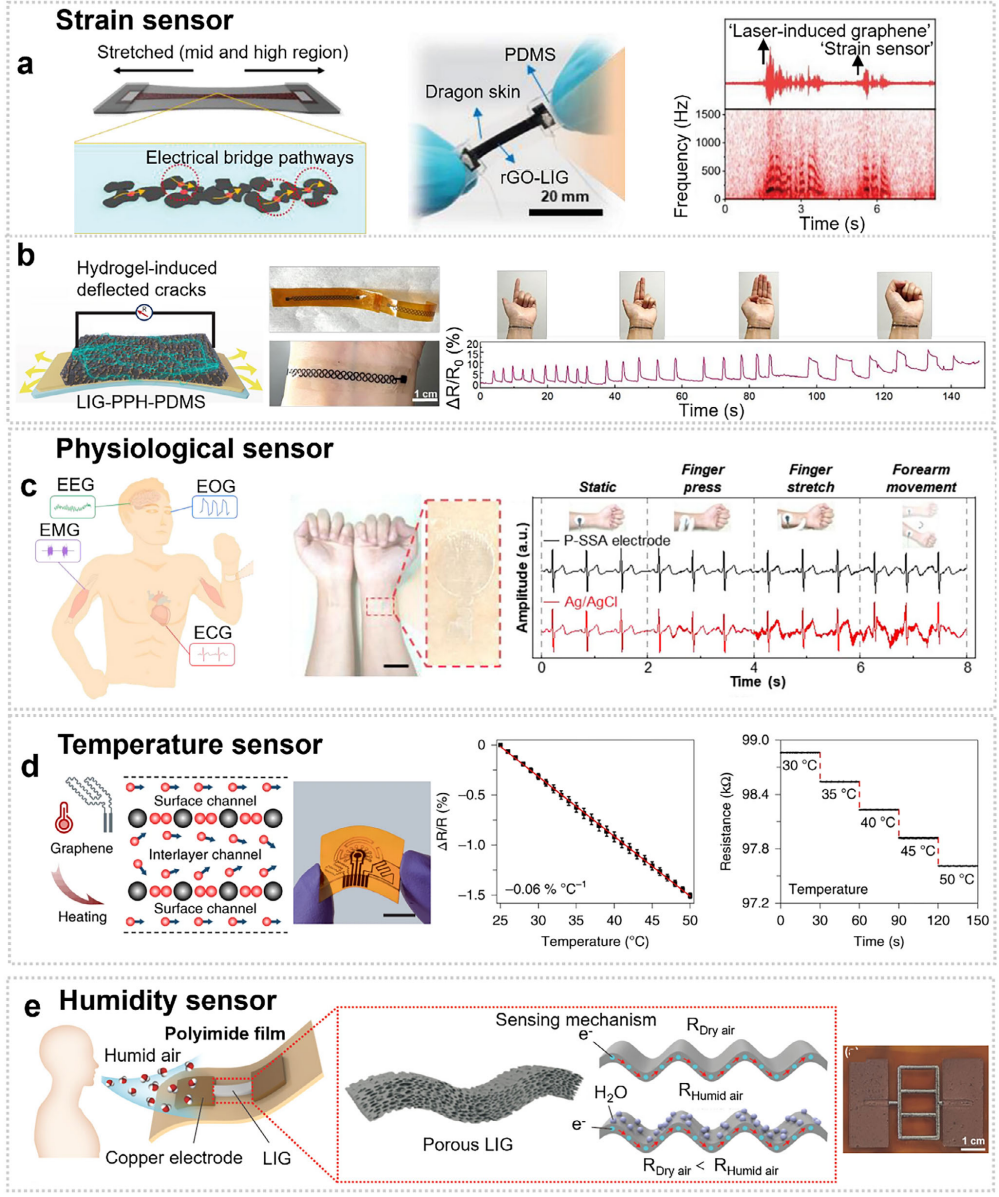
Multidimensional LIG transducers for physical biosensing. (a) Schematic illustration of the sensing mechanism of the LIG transducers‐based strain sensor. The strain sensor was mounted on the neck for acoustic vibration monitoring. Reproduced with permission [85]. Copyright 2023, Wiley‐VCH. (b) Stretchable LIG transducers‐based mechanical sensor for finger gesture recognition. Reproduced with permission [86]. Copyright 2023, Springer nature. (c) A self‐adhesive, breathable, and ultra‐thin LIG transducers‐based transferred electrode for dynamic monitoring of electrophysiological signals, such as ECG, EMG, EOG, and EEG. Reproduced with permission [91]. Copyright 2023, Wiley‐VCH. (d) Schematic illustration of the sensing mechanism of the LIG transducers‐based temperature sensor, with its calibration plot and dynamic response in physiological temperature ranges. Reproduced with permission [92]. Copyright 2019, Springer nature. (e) Schematic illustration of the sensing mechanism and a photograph of the LIG transducers‐based humidity sensor. Reproduced with permission [93]. Copyright 2024, American Chemical Society.

Mechanical deformation affects the electron transport pathways through pore dimension changes and re‐modulation of adjacent graphene nanosheet overlap within the conductive network, producing quantifiable changes in electrical resistance [74, 84, 85]. A homo‐structure reduced graphene oxide‐embedded LIG (rGO‐LIG) was fabricated by laser engraving on a graphene oxide‐polyimide composite film, followed by transferring to an elastomer, yielding an on‐body strain biosensor capable of monitoring vocal sound waveforms and other physiological movements (Figure 3a) [85]. Moreover, a cryogenically transferred LIG‐hydrogel nanocomposite with intrinsic stretchability was developed, enabling its application as a highly sensitive wearable biosensor for on‐skin gesture recognition (Figure 3b) [86]. The piezoresistive response of LIG transducers to mechanical deformation has also been leveraged for chest‐mounted respiratory monitoring, where LIG‐based sensors attached to the thorax capture the subtle mechanical deflections associated with inhalation and exhalation cycles with a high signal‐to‐noise ratio [87].

LIG fibers have also been developed as sensitive piezoresistive strain sensors by direct laser‐writing on monofilament PI fibers, achieving gauge factors of 5.43, suitable for respiratory monitoring, including inhalation and exhalation detection [13]. In addition, the laminated object manufacturing technique was employed to stack and laser‐convert PI sheets into 3D LIG foams with controllable porosity and conductivity [19]. These 3D LIG transducers were utilized as strain sensors with gauge factors of 40 and have been successfully used for pressure and pulse monitoring, achieving clear arterial pulse signal acquisition and long‐range cyclic reliability when encapsulated within elastomeric matrices. Through additive manufacturing, LIG has been developed by directly converting PI powder precursors into 3D LIG architecture with tunable geometry and mechanically robust porous networks. The resulting 3D LIG transducers incorporated into strain sensors exhibit gauge factors ranging from 78 ± 2.4 to 96 ± 2.3, demonstrating high potential for pressure and motion monitoring applications [18].

Investigations into the shapes and geometrical parameters of LIG transducers‐based strain sensors fabricated via the direct LIG fabrication on flexible PDMS substrates have achieved motion detection capabilities spanning from vocal cord vibrations to large finger and elbow joint movements, with sensitivity of GF values reaching 68 238.5 [88]. Corrugated LIG microstructures can be introduced by patterning LIG onto 3D printed PEEK substrates to achieve high gauge factors up to 2 203.5, micro‐deformation resolution of 0.01%, and durability exceeding 15 000 cycles through stress concentration and crack growth mechanisms [89]. Similarly, frustum microstructural design has been applied to LIG strain sensors by introducing two stacked and interlocked LIG transducers layers into 3D LIG structures, achieving a wide detection ranges of 0–500 kPa and a high sensitivity of 3.047 kPa^−1^ for applications in language recognition and human‐machine interaction [90].

##### 2.3.1.2 Electrophysiological Biosensing

Electrophysiological signals, such as electrocardiograms (ECGs), electromyograms (EMGs), and electroencephalograms (EEGs) provide deeper insight into the body's physiological conditions [94]. Shown in Figure 3c, functioning as dry electrodes, the LIG transducers‐integrated SEBS‐Silicone‐AgNWs (P‐SSA) device has been demonstrated, which supports long‐term monitoring of electrophysiological signals such as ECG, EMG, electrooculograms (EOG), and EEG, with applications in gesture recognition via an EMG array [91]. Furthermore, portable three‐in‐one sensory systems built using low‐impedance (<100 Ω) on‐skin LIG electrodes fabricated via direct laser writing on PI films have been developed, supporting real‐time monitoring of EEG, ECG, and EMG signals within a single integrated platform [91]. Direct laser fabrication of LIG onto Kevlar fabric enables the integration of LIG transducers with wearable textiles. LIG transducers were further combined with PEDOT:PSS coating enhancement to produce washable, mechanically robust textile ECG electrodes. These electrodes exhibit a substantial reduction in skin‐electrode impedance and produce ECG recordings with clearly resolved P waves, QRS complexes, and T waves closely matching clinical Ag/AgCl outputs. Even after repeated washing and tensile deformation cycles, the electrodes retain conductivity, structural integrity, and biocompatibility, with no observable skin irritation during extended wear [95].

##### 2.3.1.3 Temperature and Humidity Biosensing

LIG transducers enable the direct and efficient conversion of thermal gradients into measurable electrical signals, facilitating thermal sensing applications (Figure 3d) [92]. Stretchable thermoelectric composites integrating porous LIG with ionic hydrogels have been developed for self‐powered dual‐parameter sensing. These LIG/ionic hydrogel composites achieve Seebeck coefficients of −189.90 µV K^−1^, enabling temperature detection from −10°C to 100°C with 0.1°C resolution. As strain sensors, they demonstrate gauge factors up to 105.9, detection limits of 0.071%, and stability over 5000 cycles at 30% strain, suitable for applications in battery monitoring, smart garments, and medical applications [96].

Leveraging the hydrophilic surface functional groups inherent to the LIG architecture, LIG transducers provide abundant interaction sites for water molecules, enabling humidity dependent modulation of electrical conductivity. Flexible humidity sensors based on LIG transducers combined with intense pulsed light (IPL) sintering exhibit 15–92% response across 13%–67% relative humidity (RH) with selectivity up to 91.2%. These sensors successfully distinguish between normal, slow, fast, and apnea breathing patterns, serving as reliable tools for clinical respiratory monitoring and wearable health devices (Figure 3e) [93]. Integrating humidity and pressure sensing into a dual‐modality platform enables a comprehensive assessment of respiration and motion status. For instance, a dual‐mode sensor has been fabricated using a flexible composite comprising paper‐fiber‐based LIG transducers, which were post‐modified with CNTs and moisture‐sensitive hygroscopic lithium chloride (LiCl) salt. This sensor achieves pressure sensing through the piezoresistive response of the conductive carbon network and humidity sensing via ion‐mediated moisture transport within the hygroscopic salt. The device exhibits a high pressure sensitivity of 4.2 kPa^−1^, a broad linear range spanning 19.6 Pa to 39.2 kPa, and reliable operation in high‐humidity environments up to 98% RH, enabling continuous tracking of respiratory rate, skin moisture evaporation, and motion [97].

#### 2.3.2 Point‐of‐Care and Portable Biosensing

##### 2.3.2.1 LIG Transducers‐Based Electrochemical Biosensing

**FIGURE 4:**
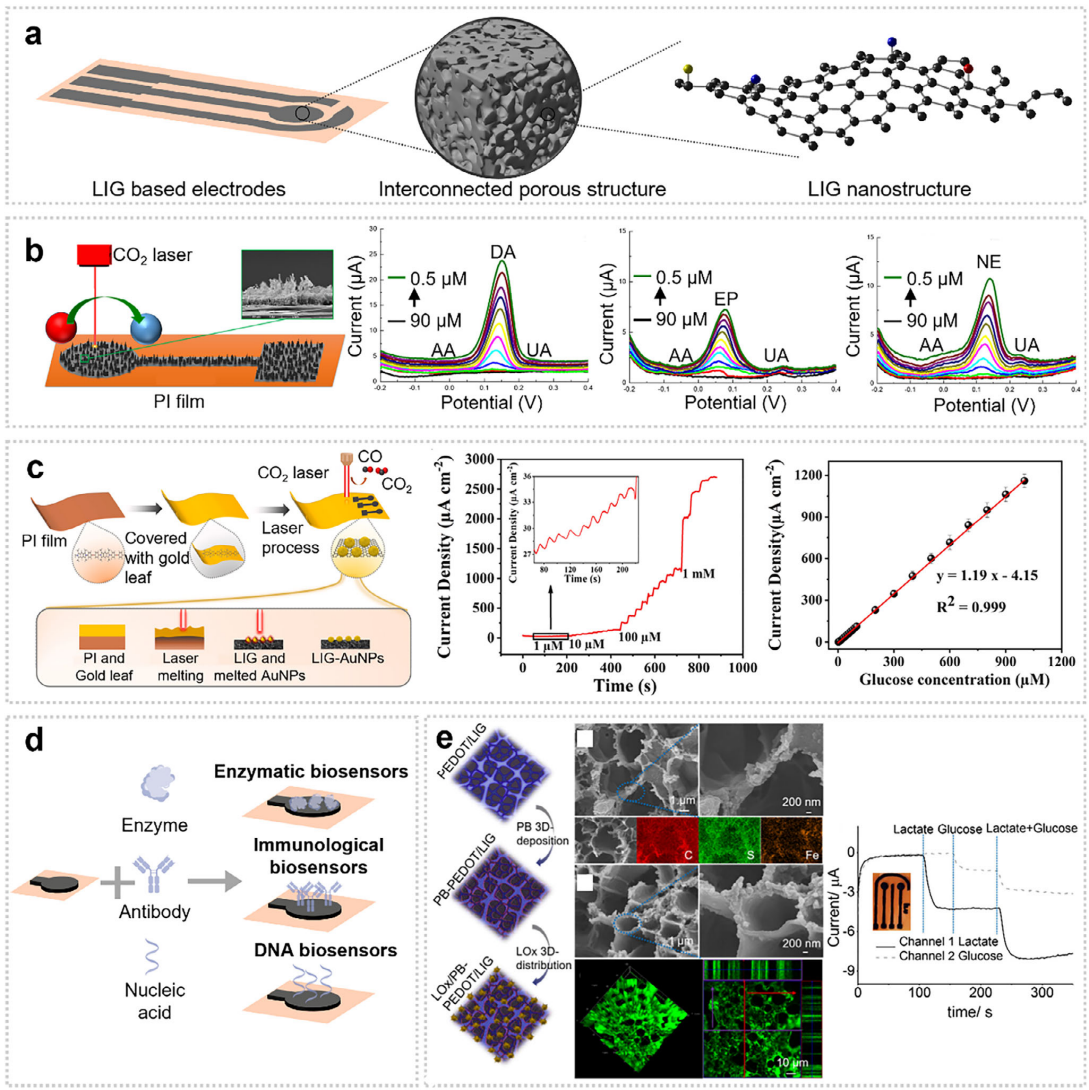
Multidimensional LIG transducers for electrochemical biosensing. (a) Schematic illustration of the porous structure of the LIG transducers. (b) Disposable LIG transducers‐based biosensor without functionalization for detecting DA, EP, and NE. Reproduced with permission [99]. Copyright 2018, American Chemical Society. (c) Schematic illustration of the synthesis of LIG‐Au nanocomposites and its application in glucose detection. Reproduced with permission [101]. Copyright 2023, Elsevier. (d) Schematic illustration of LIG transducers‐based biosensor functionalized with bioreceptors. Some figure elements were created using BioGDP.com. (e) Heterostructured 3D transducer (LOx/PB‐PEDOT/LIG) for enhancing the sensitivity, selectivity, and specificity of LIG transducers‐based lactate biosensor. Reproduced with permission [52]. Copyright 2021, American Chemical Society.

The intrinsic electrocatalytic activity of LIG transducers, arising from the graphene/graphite edge‐plane defects, oxygen‐containing functional groups, and the porous structure with high surface area, enables the electrochemical detection of electroactive small molecules without requiring additional biorecognition elements (Figure 4a) [98]. For instance, a disposable electrochemical biosensor based on porous LIG electrodes fabricated on PI films demonstrates high sensitivity and selectivity for the simultaneous detection of multiple neurotransmitters, including dopamine (DA), epinephrine (EP), and norepinephrine (NE) (Figure 4b) [99]. Furthermore, a biosensing patch based on iron nano‐catalysts incorporated LIG transducers (FeNCs/LIG) has been developed to enable highly sensitive detection of metabolites such as tyrosine (Tyr) and uric acid (UA), providing a valuable assessment of amino acid intake and metabolic status relevant to conditions including gout and kidney dysfunction [100]. Additionally, LIG transducers integrated with metal nanocomposites (e.g., Au, Ag, Pt, Pd, Cu, and AuAg alloys) on PI films have been applied to glucose biosensing, demonstrating a high sensitivity of 1194 µA mm ^−1^ cm^−2^ and reliable operation in blood plasma samples (Figure 4c) [101].

Functionalization with ion‐selective membranes extends the capabilities of LIG transducers to electrolyte monitoring, where the ion activity is translated into potentiometric signals, important for assessing hydration status, electrolyte balance, and kidney function. Biosensors with ion‐selective membranes functionalized LIG transducers have been developed to quantify sodium ions (Na^+^) with a sensitivity of 58.8 mV decade^−1^ and a linear response range of 10–100 mm [102]. Similarly, LIG transducers‐based solid‐state ion‐selective electrodes for ammonium (NH_4_^+^) and potassium (K^+^) ions detection in urine have been fabricated to assess hydration status. These sensors achieve detection limits of 30 µm for ammonium and 100 µm for potassium, with high stability over three months and rapid 30‐s response times [103]. LIG transducers incorporating laser‐printed copper and cuprous oxide nanomaterials have been developed for noninvasive kidney function monitoring via sweat analysis of K^+^, creatinine (Cre), and lactic acid (Lac). This portable system integrate two‐ and three‐electrode configurations with printed circuit board workstations, demonstrating good sensitivity and selectivity for chronic kidney disease (CKD) biomarkers [104].

##### 2.3.2.2 Biofunctionalized LIG Transducers‐Based Biosensing

The biofunctionalization with biorecognition elements confers molecular specificity to the LIG transducers, enabling selective detection of target analytes within the complex biological matrices such as sweat, blood, saliva, and interstitial fluid (Figure 4d) [11].

Immobilization of enzymes onto LIG electrodes enables highly selective detection of specific molecules through enzyme‐catalyzed reactions that generate electrochemically detectable products. Chitosan‐glucose oxidase (GOx) composites immobilized onto acetic acid‐modified LIG electrodes enable selective sweat glucose detection with a high sensitivity of 4.622 µA mm ^−1^ and a dynamic linear range up to 2.1 mm [105]. Similarly, PEDOT‐reinforced LIG electrodes were functionalized with Prussian blue and immobilized with lactate oxidase to develop a flexible skin patch lactate biosensor, with a high sensitivity (11.83 µA mm ^−1^) and selectivity toward various interfering molecules such as glucose, ascorbic acid, creatinine, and uric acid (Figure 4e) [52]. To fabricate disposable electrodes in a low‐cost and sustainable manner, Mak's team fabricated green route lignin‐derived 2D LIG electrodes (P‐rLIG). The P‐rLIG electrodes was deposited and functionalized with lactate oxidase, delivering a high analytical performance with a linear range up to 16 mm, a high sensitivity of 1.21 µA mm ^−1^, and a selectivity toward various potentially interfering materials such as ascorbic acid (AA), UA, and creatinine [53]. 3D LIG patches fabricated by multilayer stacking of laser‐engraved LIG patterns substantially enhance integration of multiple sensing modalities. The biosensing layer in such devices incorporates polyaniline (PANI)‐based electrodes for reversible pH monitoring (sensitivity ∼59 mV/pH across pH 4–10) and poly(3,4‐ethylenedioxythiophene) (PEDOT)/Prussian Blue/uricase‐functionalized electrodes for pH‐compensated uric acid detection (linear range 0–0.9 mm) [34].

Antibody‐functionalized LIG electrodes enable the sensitive and specific detection of protein biomarkers through antigen‐antibody binding interactions. Multiplex LIG immunosensors for tumor marker detection, including neuron‐specific enolase (NSE), carcinoembryonic antigen (CEA), p53, and SOX2 have been developed to support early lung cancer screening. Combined with deep learning‐based computed tomography (CT) imaging and clinical data, multimodal predictive models have been established for comprehensive early lung cancer screening protocols [106]. Self‐assembled monolayers (SAMs) of 3‐mercaptopropionic acid immobilized on gold nanoparticle (AuNP)‐functionalized LIG transducers are activated for binding anti‐cortisol antibodies. The resulting biosensors exhibit specific cortisol detection over the physiologic range with a LOD of 0.0085 nm [107]. Multiplexed immunosensing platforms built on LIG transducers have also been developed to detect viral antigens, antibodies, and inflammatory markers via amperometric measurements for telemedicine‐based COVID‐19 diagnostics [108]. Ultrasensitive detection (1 Hz pg^−1^ mL^−1^ frequency shift) of the SARS‐CoV‐2 spike protein has been achieved through direct antibody immobilization onto porous LIG transducers surfaces. Capitalizing on inherent nanoscale porosity and high electrical conductivity, remarkable frequency shifts are realized in impedance‐based platforms, enabling sub‐minute response times and empowering early outbreak containment through decentralized testing [109].

Aptamers, which are single‐stranded DNA or RNA oligonucleotides that bind targets with high affinity and specificity, offering advantages including chemical stability, ease of synthesis, and regenerability. Thrombin detection has been demonstrated using aptamer‐functionalized LIG electrodes, establishing foundational approaches for LIG‐based aptamer sensors [110]. LIG‐based extended‐gate field‐effect transistors (LIG‐EG‐FETs) functionalized with aptamer layers have been developed for selective vancomycin detection with an LOD of 0.187 nm. In this configuration, LIG transducers are patterned with MnO_2_ nanoparticles as MnO_2_/LIG electrodes following by AuNPs loaded onto working electrode regions enhance electrical performance and provide ideal binding sites for sulfhydryl‐modified aptamers. Vancomycin aptamers linked to electrodes via Au–S coordination form SAMs, enabling specific binding with the target antibiotic [111]. Modified with DNA probes, LIG microelectrode arrays have been constructed for multiplex miRNA detection within microfluidic platforms. Utilizing enzyme‐assisted target recycling amplification, these LIG arrays detect miRNA expression levels in both exosomes and clinical serum samples, opening new avenues for early cancer diagnosis using routine clinical specimens [65]. LIG electrodes incorporating AuNPs and antibody recognition layers have been demonstrated to selectively detect N^6^‐methyladenosine (m^6^A) RNA and 5‐methylcytosine (5mC) DNA, delineating the potential of immunorecognition approaches in epigenetic nucleic acid analysis [112].

#### 2.3.3 Wearable and Implantable Biosensing for Personalized Health Monitoring

Wearable biosensors enable the real‐time monitoring of biological signals such as heart rate, glucose levels, and biomarkers in biofluid, playing a pivotal role in personalized healthcare, chronic disease management (e.g., diabetes, cardiovascular conditions, and cancer), and fitness optimization [113]. For example, sweat, as a rich source of metabolic and electrolyte biomarkers accessible non‐invasively at the skin surface, is a particularly attractive biofluid for wearable biochemical monitoring [114]. By continuously capturing physiological data in nature, these devices empower individuals to track their unique health trajectories and enable clinicians to tailor interventions based on patient‐specific patterns rather than population averages.

##### 2.3.3.1 Multimodal Wearable Biosensors

Wearable sensors designed to monitor individual physiological parameters have laid the foundation for more complicated multimodal systems. Human physiological activities, including body movements, biochemical metabolism, and vital signs, are inherently complex and multifaceted, necessitating a comprehensive and tailored health assessment through simultaneous monitoring of multiple physiological parameters.

**FIGURE 5:**
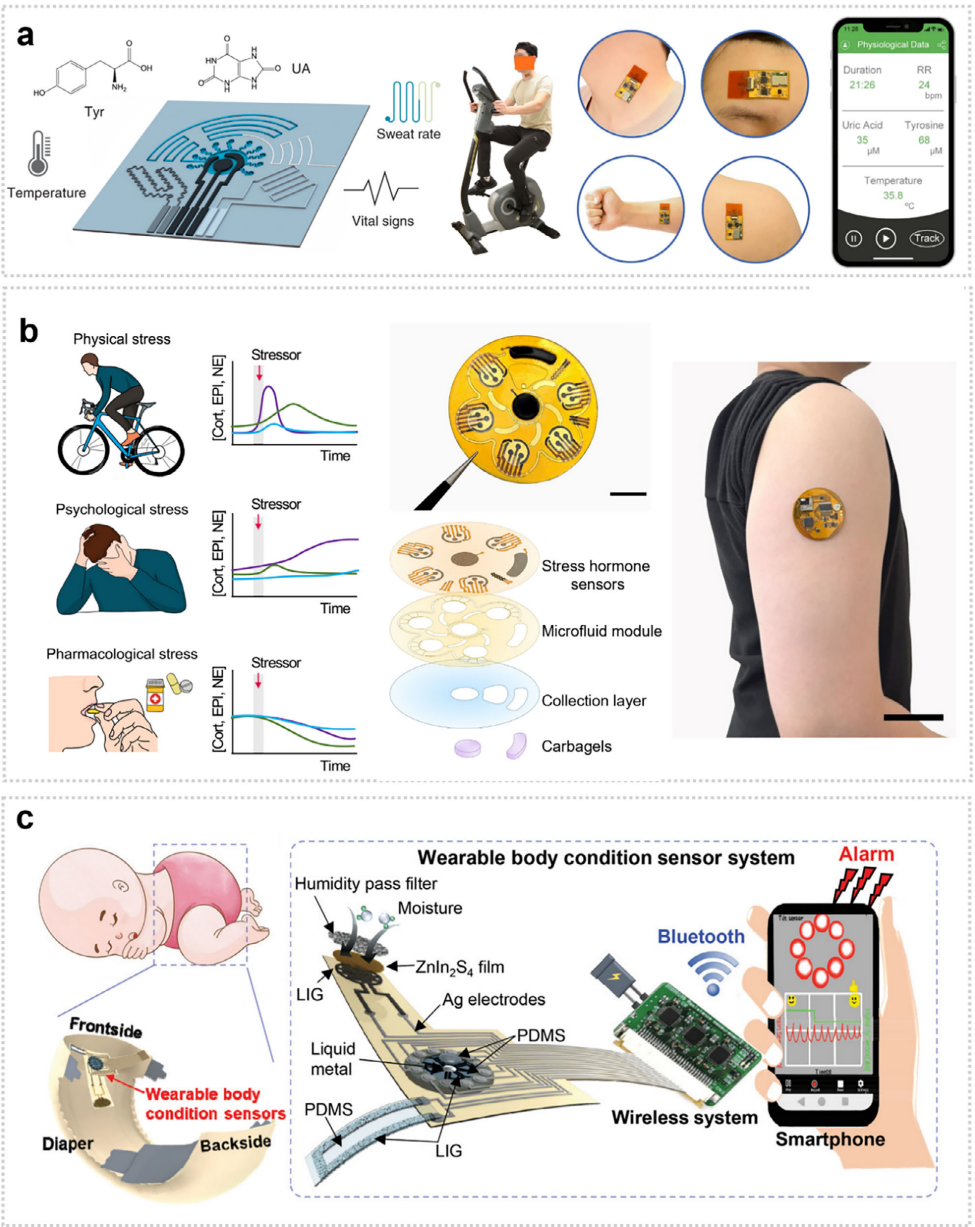
Multidimensional LIG transducers for multimodal wearable sensing. (a) A multifunctional LIG transducers‐based wearable biosensor for sweat UA and Tyr detection, sweat rate estimation, temperature sensing and vital‐sign (e.g., heart rate, respiration rate) monitoring. Reproduced with permission [92]. Copyright 2019, Springer nature. (b) Schematic illustration of a microfluidic integrated LIG transducers‐based mobile biosensor for sweat cortisol, epinephrine, and NE monitoring. Reproduced with permission [115]. Copyright 2025, American Association for the Advancement of Science. (c) Schematic illustration of a wearable sensor system attached to a disposable diaper for vulnerable population monitoring. Reproduced with permission [116]. Copyright 2021, Wiley‐VCH.

To facilitate personalized analysis of gout metabolism, multimodal biosensing systems have been developed, consisting of highly sensitive LIG transducers‐based chemical sensors (LEG‐CS) for monitoring low concentrations of uric acid (UA) and tyrosine (Tyr), multiplexed LIG transducers‐based physical sensors (LEG‐PS) for monitoring temperature and respiration rate, and laser‐engraved multi‐inlet microfluidic modules for dynamic sweat sampling (Figure 5a) [92]. By establishing individual baseline profiles and tracking deviations over time, such platforms allow patients to receive dietary and lifestyle guidance calibrated to their specific metabolic responses rather than generic clinical thresholds. A wearable multimodal microfluidic biosensor device employing gold nanodendrite‐decorated LIG electrodes has been demonstrated for simultaneous detection of cortisol, epinephrine, and norepinephrine, enabling noninvasive, real‐time stress management, early detection of maladaptive responses, and theranostic applications in personalized medicine. Through integrating iontophoresis‐driven sweat extraction with capillary valve‐regulated microfluidic channels for automated sampling, reconstitution, and reagent delivery, this device reliably tracks hormone fluctuations in response to physical (e.g., HIIT‐induced cortisol and norepinephrine spikes), psychological (e.g., IAPS‐triggered norepinephrine elevations), and pharmacological stressors (e.g., supplement‐mediated cortisol reductions) (Figure 5b) [115]. By combining tilt, strain, and humidity responses within a wearable body condition sensor system, versatile LIG transducers‐based wireless e‐textile platforms have been developed for simultaneously tracking sleeping posture, respiration rate, and diaper moisture in infants. This all‐in‐one, battery‐powered device eliminates cumbersome wiring, delivers real‐time actionable insights tailored to each individual's developmental stage or health condition to caregivers, and significantly mitigates risks such as sudden infant death syndrome, demonstrating the broad personalized healthcare potential of LIG transducers‐enabled multimodal biosensing in neonatal and geriatric care (Figure 5c) [116].

These examples illustrate the versatility of LIG transducer‐based multimodal wearable biosensors in addressing diverse healthcare needs, from metabolic disorder management to stress monitoring and neonatal care. By integrating multiple biosensing modalities, including physical and biochemical biosensing, within a single platform, these systems capture the multifaceted nature of human health and enable personalized assessment that adapts to individual baselines and lifestyles. As research in this field continues to expand, LIG transducers‐enabled multimodal biosensors are being tailored for an increasingly broad spectrum of applications, including chronic disease management, wound healing assessment, nutritional optimization, fitness tracking, and sports medicine. Table 2 provides a comprehensive overview of representative integrated LIG transducers‐based multimodal biosensor systems, categorized by healthcare applications spanning cardiovascular monitoring, metabolic disorders, respiratory monitoring, oncology, infectious disease detection, neuroscience, and motion rehabilitation. The table highlights the diverse LIG‐based platforms, sensing modalities (physical, biochemical, physiological, and multimodal), target analytes or parameters, and underlying sensing mechanisms (e.g., piezoresistive, amperometric, voltammetric, potentiometric, impedimetric) that collectively enable personalized health monitoring and disease management.

**TABLE 2:** Representative integrated LIG transducers‐based multimodal biosensor systems.

Healthcare application	LIG‐based platform	Sensing modality	Target Analyte/Parameter	Sensing Mechanism	Refs.
Cardiac monitoring	LIG‐PPH‐PDMS	Physiological	ECG	Capacitive/Resistive	[86]
	PEDOT:PSS/LIG	Physiological	ECG	Resistive	[95]
	LIG/LCP	Physiological	ECG, EMG, ECoG	Resistive	[58]
	LIG/PI	Physiological	EEG, ECG, EMG	Resistive	[117]
	Co‐NPC@LIG	Physical	Pulse, arterial waveform	Piezoresistive	[84]
	LIG/PEEK	Physical	Pulse	Piezoresistive	[89]
Personalized metabolic care	LIG‐Au nanocomposite	Biochemical	Glucose	Amperometric	[101]
	LIG/GOx	Biochemical	Glucose	Amperometric	[102]
	LIG/PtNPs/GOx	Biochemical	Glucose	Amperometric	[105]
	LOx/PB‐PEDOT/LIG	Biochemical	Lactate	Amperometric	[52]
	LOx/PB/P‐rLIG	Biochemical	Lactate	Amperometric	[53]
	FeNCs/LIG	Biochemical	Tyr, UA	Voltammetric	[100]
	LIG/PI	Multimodal	UA, Tyr, temperature, respiration rate	Voltammetric/Resistive	[92]
Kidney function monitoring	LIG/PEDOT/ISM, LIG/Cu	Biochemical	K^+^, Creatinine, Lactate	Potentiometric/Amperometric	[104]
	LIG/ISM	Biochemical	NH_4_^+^, K^+^	Potentiometric	[103]
Stress & mental health management	LIG/AuNPs/antibody	Biochemical	Cortisol, EPI, NE	Amperometric	[115]
	LIG/antibody	Biochemical	Cortisol	Amperometric	[118]
	LIG/AuNP	Biochemical	Cortisol	Impedimetric	[107]
Respiratory monitoring	LIG‐CuNPs	Physical	Humidity	Resistive	[93]
	LIG/PI	Physical	Respiration (strain)	Piezoresistive	[13]
	LiCl‐Graphene‐CNT‐paper fiber	Physical	Humidity, strain	Resistive/Piezoresistive	[97]
	LIG/PI	Physical	Respiration (strain)	Piezoresistive	[87]
Precision medicine in oncology	LIG/antibody	Biochemical	CEA, NSE, p53, SOX2	Amperometric	[106]
Infectious disease detection	LIG/antibody	Biochemical	SARS‐CoV‐2 antigen, antibodies, CRP	Amperometric	[108]
	Freestanding LIG flakes	Biochemical	SARS‐CoV‐2 spike protein	Impedimetric	[109]
Neuroscience study & BCI	LIG/PI	Physiological	EEG, ECoG, neural spike	Resistive	[119]
Motion detection& rehabilitation	rGO‐LIG	Physical	Strain (gesture, movement)	Piezoresistive	[85]
	LIG/PDMS	Physical	Strain (motion)	Piezoresistive	[88]
	AgNWs/LIG	Multimodal	EMG (gesture recognition)	Resistive	[91]
Neonatal & geriatric care	LIG/liquid metal/PDMS	Multimodal	Tilt, strain, humidity	Resistive/Capacitive	[116]
Fitness & sports medicine	LIG/GOx, LIG/LOx, LIG/ISM	Biochemical	Glucose, lactate, Na^+^	Amperometric/Potentiometric	[102]
	LIG/silicone elastomer	Multimodal	EEG, ECG, EMG, hydration, temperature	Resistive/Thermoelectric	[22]
	NKKC/LIG	Multimodal	Temperature, strain	Thermoelectric/Piezoresistive	[96]
Point‐of‐care diagnostics	LIG/PI	Biochemical	DA, EP, NE	Voltammetric	[99]
	LIG/PI	Biochemical	AA, DA, UA	Voltammetric	[98]
	LIG/aptamer	Biochemical	Thrombin	Impedimetric	[110]
	LIG/aptamer	Biochemical	Vancomycin	FET‐based	[111]
	LIG/AuNPs/antibody	Biochemical	m^6^A‐RNA, 5mC‐DNA	Amperometric	[112]

Abbreviations:

AA: Ascorbic acid; AgNWs: Silver nanowires; AuNP(s): Gold nanoparticle(s); BCI: Brain‐computer interface; CEA: Carcinoembryonic antigen;

CNT: Carbon nanotube; Co‐NPC: Cobalt nanoporous carbon; CRP: C‐reactive protein; DA: Dopamine;

ECG: Electrocardiography; ECoG: Electrocorticography; EEG: Electroencephalography; EMG: Electromyography;

EP/EPI: Epinephrine; FeNCs: Iron‐based nano‐catalysts; FET: Field‐effect transistor; GO: Graphene oxide;

GOx: Glucose oxidase; ISM: Ion‐selective membrane; LCP: Liquid crystal polymer; LIG: Laser‐induced graphene;

LOx: Lactate oxidase; NE: Norepinephrine; NKKC: NaCl/K_3_[Fe(CN)_6_]/K_4_[Fe(CN)_6_]/CMC Na ion gel;

NSE: Neuron‐specific enolase; PB: Prussian blue; PDMS: Polydimethylsiloxane; PEDOT:PSS: Poly(3,4‐ethylenedioxythiophene):poly(styrene sulfonate);

PEEK: Polyether ether ketone; PI: Polyimide; PPH: Polyvinyl alcohol‐phytic acid‐honey; PtNPs: Platinum nanoparticles;

PU: Polyurethane; rGO: Reduced graphene oxide; SOX2: SRY‐box transcription factor 2; Tyr: Tyrosine; UA: Uric acid.

##### 2.3.3.2 Self‐Powered Wearable Biosensors

Continuous wearable monitoring demands sustainable power solutions that minimize battery dependence while maintaining device functionality. A direct laser writing process for generating LIG electrodes on Kevlar textiles has been demonstrated, yielding the anisotropic bifacial structures with conductive graphene patterns on the mechanically durable Kevlar textile. This configuration supports multifunctional device integration, including Zn–air batteries, ECG electrodes, and NO_2_ gas sensors, establishing a rapid and versatile route toward intelligent protective garments with embedded sensing and energy‐harvesting functions [79].

LIG transducers‐based self‐powered wearable strain sensors have been realized by constructing layered architectures of LIG transducers on rGO‐cloth substrates. The layered configuration of the LIG transducers significantly boosts sensor sensitivity from 20.6 kPa^−1^ to 30.3 kPa^−1^ while simultaneously enhancing the transferred charge density of integrated triboelectric nanogenerators (TENGs) from 160 µC m^−2^ to 270 µC m^−2^. These LIG transducers enable the fabrication of completely self‐powered, battery‐free systems that integrate pressure sensing and energy‐harvesting on the flexible textile platform. The devices are energy‐efficient, highly sensitive, and capable of detecting subtle human motions and static forces [120]. Foldable and LIG TENGs fabricated on PI papers enable stable origami‐like multi‐layer structures that boost open‐circuit voltage from 5.3 to 34.4 V cm^−2^. This structure enables devices with conformal, battery‐free wearable biosensing that accurately recognize walking and running gaits in smart shoes, distinguish grasped objects and finger contacts via smart gloves, and capture real‐time handwriting trajectories and complex touch patterns through palm‐attached matrix sensors [74].

##### 2.3.3.3 Implantable Biosensing Systems

**FIGURE 6:**
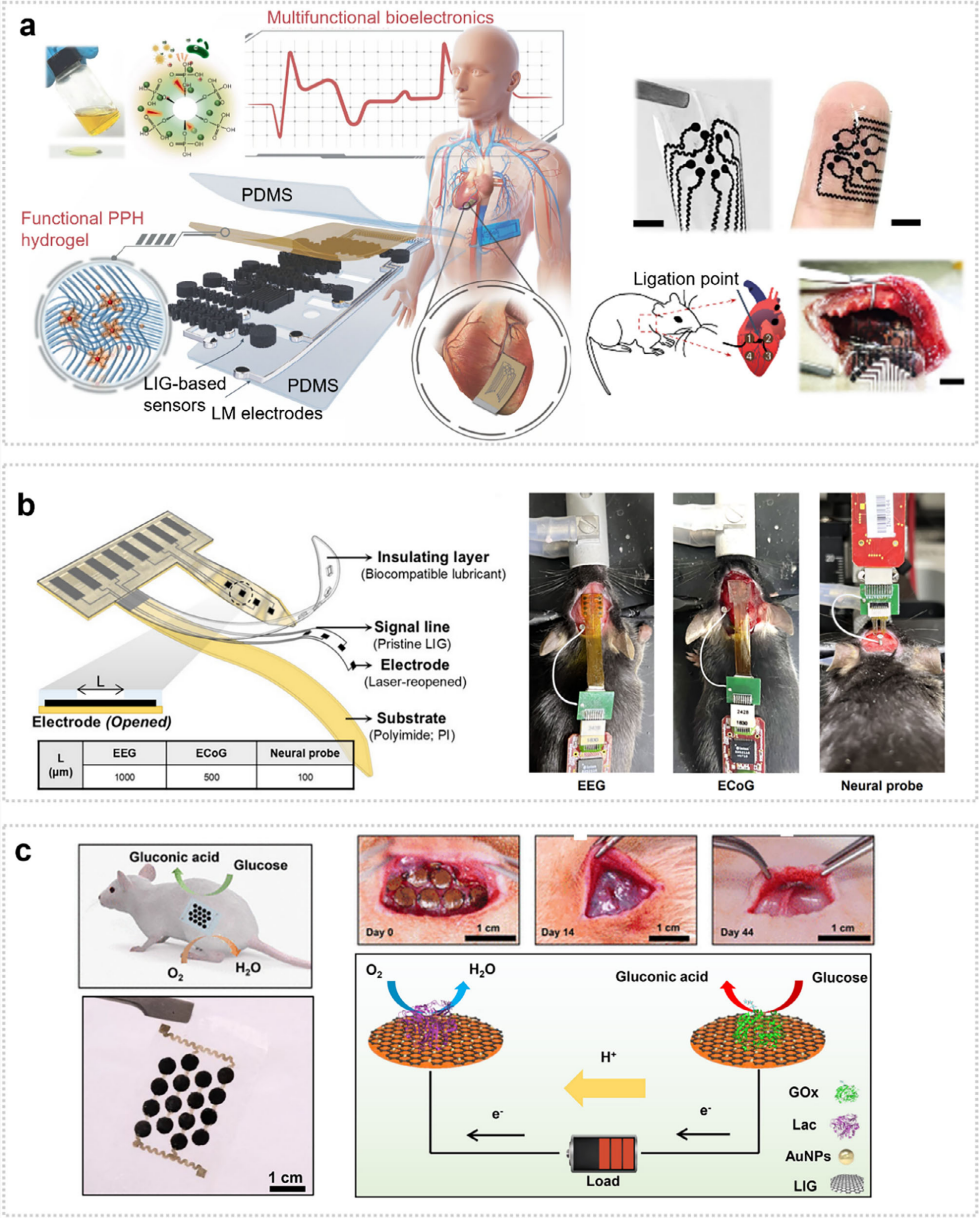
Multidimensional LIG transducers for implantable biosensing. (a) Schematic illustration of a thin, antibacterial, and biocompatible transferable LIG transducers‐based PPH hydrogel‐enhanced stretchable nanocomposites for wearable and implantable biosensors. Reproduced with permission [86]. Copyright 2024, Springer nature. (b) Structure of a biocompatible implantable biosensor for in vivo EEG, ECoG, and spike signals recording. Reproduced with permission [119]. Copyright 2025, Wiley‐VCH. (c) Implantable transient glucose enzymatic biofuel cells based on LIG transducers. Reproduced with permission [59]. Copyright 2022, American Chemical Society.

Implantable biosensors are medical devices inserted into the body to provide continuous, real‐time monitoring of physiological data like glucose levels or pressure [121]. An ultrathin, highly stretchable LIG–hydrogel conductive composite has been developed via a cryogenic transfer process that dramatically strengthens interfacial bonding and transforms brittle LIG into an intrinsically stretchable conductor capable of exceeding 200% strain. The LIG–hydrogel composite has been successfully deployed as a conformal four‐channel epi‐cardial patch on rat hearts with high mechanical compliance and long‐term signal quality in vivo for real‐time diagnosis and continuous monitoring of cardiac pathologies in fully implantable settings (Figure 6a) [86]. Customizable LIG transducers‐based neural electrodes, including EEG, electrocorticography (ECoG), and penetrating probes, have been demonstrated. In vivo recordings in mice demonstrate that these LIG electrodes capture stable EEG and ECoG signals across the full frequency spectrum and detect neural spikes with high fidelity, while maintaining favorable impedance and cell viability in biocompatibility assays. The rapid, low‐cost LIG fabrication route makes it feasible to tailor electrode geometries and channel densities to specific patients or animal models, supporting personalized neurodiagnostics and brain‐computer interface development (Figure 6b) [119]. LIG transducers can also support implantable power sources that match the softness and degradable requirements of bioelectronic implants. Transient enzymatic LIG transducers‐based biofuel cells have been developed based on LIG/gold nanoparticle composite electrodes, patterned on PI films and transfer‐printed onto biodegradable poly(lactic‐co‐glycolic acid) (PLGA) substrates. The electrodes exhibit low charge–transfer resistance (∼16 Ω), enabling high‐performance glucose biofuel cells with an open‐circuit potential of 0.77 V and a maximum power density of 483.1 µW cm^−2^, suitable for powering low‐power implantable medical electronics. The device adheres well to curved organ surfaces and demonstrates good in vitro and in vivo biocompatibility, operating for over 28 days before gradually degrading. By harvesting biochemical energy from physiological glucose, such LIG transducers‐based transient power modules can be combined with LIG sensors and neural interfaces to create integrated, self‐powered implantable systems that minimize secondary surgeries and align with the goals of patient‐specific, time‐limited therapies (Figure 6c) [59].

## 3 LIG‐Based Platforms for Multifunctional Biomedical Applications Toward Personalized Healthcare

The application versatility of the LIG extends beyond biosensing into multifunctional biomedical systems. LIG supports the establishment of bioactive interfaces, including antimicrobial protection, therapeutic drug delivery, and microfluidic chip integration. Furthermore, LIG‐based platforms enable regenerative medicine applications, including tissue regeneration scaffolds and cell‐stimulating substrates, alongside nucleic acid amplification for molecular diagnostics.

### 3.1 LIG‐Based Bioactive Interfaces with Anti‐Microbial Capabilities for Wearable Clinical Applications

Long‐term application of wearable biosensors on the skin or within wounds faces challenges, including bacterial colonization and biofilm formation that degrade sensor performance and elevate infection risks [122], particularly in clinical applications such as chronic wound management [123], postoperative monitoring [124], and transdermal drug delivery [125]. Owing to its porous architecture and tunable surface chemistries, LIG can simultaneously serve as a functional transducer and an antimicrobial interface, enhancing electrochemical biosensing performance while resisting microbial adhesion and disrupting biofilm formation.

The microstructure of LIG can be tuned into the interconnected open‐well architecture with high specific surface areas exceeding 170 m^2^ g^−1^ as trapping sites that exhibit potent bactericidal effects. Such designed microstructures have demonstrated the ability to eliminate approximately 93% of *E. coli* and 95% of *S. aureus* through combined membrane stress and oxidative damage mechanisms [33]. The surface of LIGs can also be engineered to a nanofibrous architecture through topographical design to prevent biofilm formation. The vertically aligned texture of the nanofibrous LIG architecture suppresses biofilm accumulation of both rod‐shaped bacteria (such as *E. coli*) and coccus‐shaped species (such as *Staphylococcus epidermidis*) over extended exposure periods exceeding 10 days [126]. Bacteria encountering these nanofibrous surfaces exhibit reversible morphological stress, including cell‐size reduction, without permanent genetic alterations, indicating that LIG can mechanically and topographically disrupt early adhesion and biofilm maturation. Surface chemistry modification of LIG offers another avenue for passive antimicrobial protection. Through covalently grafting quaternary pyridinium groups onto the LIG surface, the native slightly negative surface can be converted into a strongly positively charged coating, which enhanced the electrostatic attraction and negatively charged pathogen membrane disruption. Combined with LIG's intrinsic photothermal response under the ambient light, leading to the rapid pathogen inactivation without leaching biocidal agents. This dual mechanism enables fast, broad‐spectrum pathogen removal under mild conditions [127].

LIG transducers‐based electrical actuation also enables on‐demand biofilm disruption. Applying modest electrical potentials across LIG electrodes in contact with electrolyte can rapidly disrupt and remove established biofilms without requiring added chemicals [128]. By integrating such electrodes beneath sensing sites, wearable devices could periodically self‐clean their interfaces through brief electrical pulses, restoring signal quality and reducing contamination during extended monitoring campaigns. By combining the photothermal activation function, LIG transducers‐based electrodes can be integrated into commercial masks, offering a complementary, wireless approach to on‐demand antimicrobial protection [61]. This concept has been further extended through flexible LIG composite adhesive tapes that combine Joule heating with light‐induced heating to achieve synergistic electro‐photo‐thermal antimicrobial effect without damaging the substrate, highlighting their suitability as protective outer layers or edge seals on wearable theranostic patches [62]. Collectively, these antimicrobial strategies demonstrate the potential of LIG as a bioactive interface that reacts to environmental stimuli and actively modulates the local biological environment to reduce the risk of infection.

### 3.2 LIG Transducers‐Based Bioactive Drug Delivery Platforms for Smart Theranostics Applications

LIG transducers‐based platforms also enable closed‐loop theranostic where real‐time health assessment directly informs and triggers therapeutic action. The porous architecture and conductive network of LIG transducers enable sufficient drug loading, while the high electrical conductivity and photothermal transduction capabilities enable controlled drug release.

**FIGURE 7:**
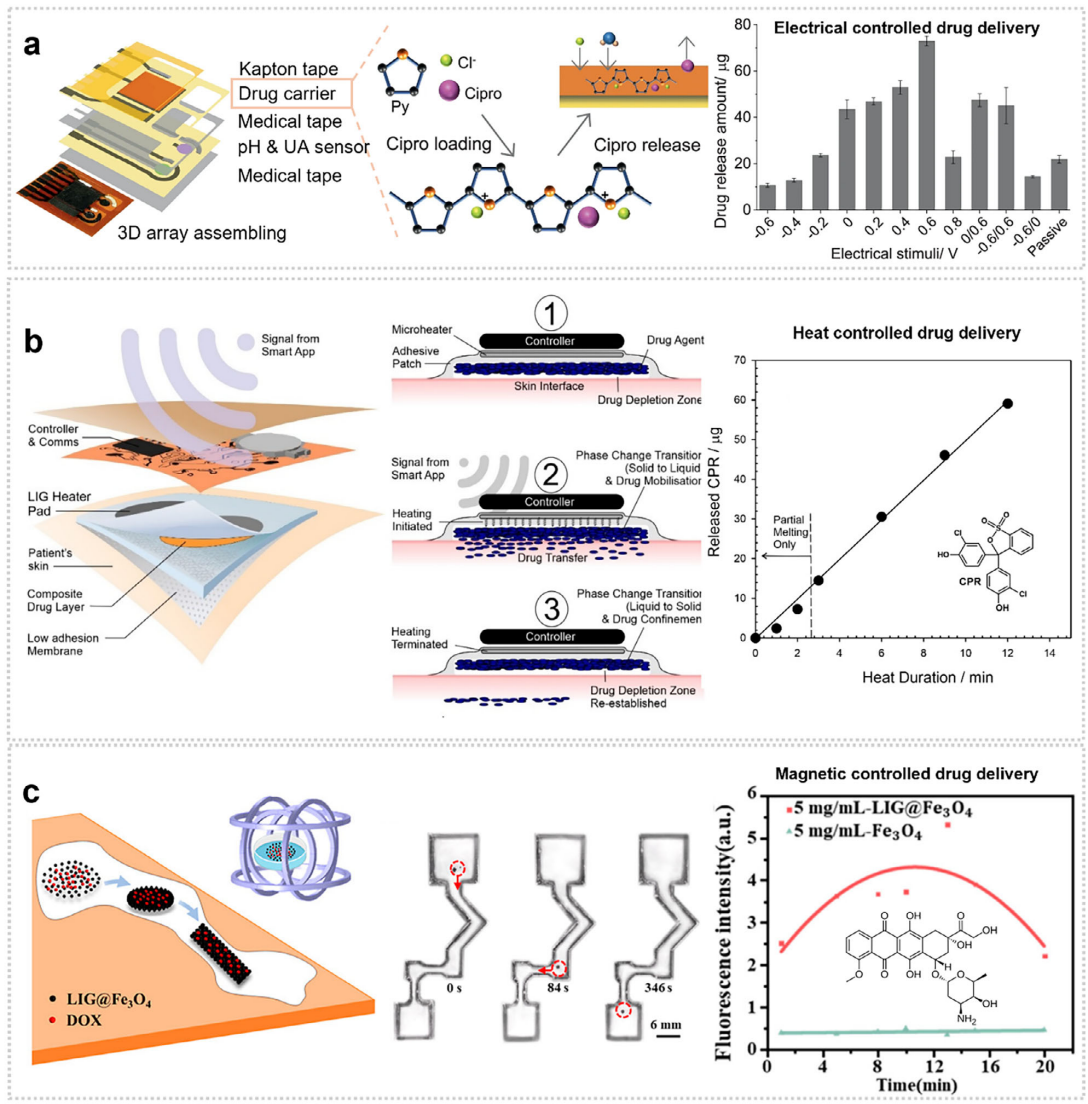
LIG transducers‐based drug delivery platforms. (a) Schematic illustration of a LIG transducers‐based active drug delivery patch. Reproduced with permission [34]. Copyright 2024, Springer nature. (b) Schematic illustration of an electronically controlled drug delivery patch with a LIG transducers‐based heater. Reproduced with permission [129]. Copyright 2022, MDPI. (c) Schematic illustration of porous LIG transducers‐based nanorobots for site‐specific targeted drug delivery. Reproduced with permission [68]. Copyright 2024, American Chemical Society.

Mak's team recently developed a LIG transducers‐based smart bandage for chronic wound care, combining multiplex sensing with electrically triggered antibiotic delivery (Figure 7a) [34]. The system integrates LIG‐polyaniline (PANI) electrodes for reversible pH monitoring (sensitivity ∼59 mV/pH across pH 4–10) and functionalized LIG/PEDOT/Prussian Blue/uricase electrodes for pH‐compensated uric acid sensing (linear range 0–0.9 mm). The drug release component employs LIG‐polypyrrole (PPy) electrodes for on‐demand ciprofloxacin release actuated by a low 0.6 V stimulus. Assembled into a stacked patch with wireless connectivity, this device continuously assesses wound conditions and delivers on‐demand antimicrobial doses only when needed, reducing infection risk. LIG transducers‐based microheaters have also been integrated with phase‐change materials to realize therapeutic drug delivery (Figure 7b) [129]. Curcumin was encapsulated within a phase‐change matrix that remains solid under ambient conditions. Upon the electrical activation of underlying LIG microheaters, localized Joule heating melts the matrix and releases a defined dose before re‐solidification halts further diffusion. This approach enables controllable and patient‐specific dosing schedules synchronized with physiological readouts or external programming.

Besides drug delivery patches, LIG transducers‐based magnetic nanorobots have also been developed for targeted drug delivery to deep tissue sites (Figure 7c) [68]. The LIG‐Fe_3_O_4_ particles with approximately 50‐fold higher doxorubicin loading capacity than bare Fe_3_O_4_ magnetic nanoparticles retain ∼96% of their cargo while navigating complex microfluidic geometries. Magnetic actuation enables navigation through artificial physiological environments, while near‐infrared light triggers photothermal release at the target site. This spatiotemporally controlled activation minimizes off‐target toxicity, pointing toward minimally invasive theragnostic systems capable of real‐time guidance and adaptive dosing at targeted disease sites.

### 3.3 LIG Transducers Integrated Lab‐on‐a‐Chip (LOC) Platforms

The tunable wettability of LIG transducers enables microfluidic device fabrication with pump‐free fluid manipulation. By varying LIG laser fabrication parameters, superhydrophilic channels bounded by superhydrophobic rails can be fabricated on PI substrates to enable capillary‐driven flow that passively splits the sample into multiple branches to feed independent LIG transducers‐based ion‐selective electrodes and enzymatic biosensors [35]. This design realizes multiplexed detection of ions and pesticides at picomolar levels without external pumps. LIG transducers can also be embedded into microfluidic channel structures to realize integrated lab‐on‐a‐chip (LOC) devices where sample routing, reaction, and readout occur within a single cartridge. An adhesive‐tape bonded LOC has been demonstrated for pathogen detection, with LIG working, reference, and counter electrodes aligned with microfabricated microfluidic channels. Liposome‐amplified electrochemiluminescent and electrochemical assays of *Cryptosporidium* DNA achieved LOD of 3 pm and 47 pm, respectively, demonstrating the highly sensitive nucleic‐acid biosensing in a compact, low‐cost platform suitable for point‐of‐care devices [130].

LIG transducers‐based LOC on cellulose papers was also demonstrated. LIG electrodes embedded within the paper matrix retain their natural hydrophilicity enable efficient capillary‐driven flows [21]. These paper‐based LIG transducers‐based devices have been fabricated into lateral and vertical flow configurations for the alkaline phosphatase detection in serum and human papillomavirus (HPV) detection using CRISPR‐based assays. The combination of electrochemical biosensing with paper microfluidics offers a scalable, sustainable, and low‐cost approach for disease diagnostics and environmental monitoring.

### 3.4 LIG Microheaters for Nucleic Acid Amplifications

LIG transducers further extend LOC platforms by providing the precise thermal control necessary for on‐chip molecular diagnostics. The Joule heating ability of LIG transducers makes them well‐suited for microheater fabrication to enable nucleic acid amplifications such as polymerase chain reaction (PCR). A microfluidic PCR device utilizing LIG microheaters has demonstrated rapid thermal cycling with heating rates of 82°C per minute [37]. The device successfully amplified a 404 base pair DNA target from *Porphyromonas gingivalis* over 36 thermal cycles, validating LIG microheaters as effective thermal elements for portable PCR diagnostics. LIG microheaters also offer simplified thermal management by maintaining a constant temperature. An integrated on‐chip system combining LIG microheaters for loop‐mediated isothermal amplification (LAMP) with CRISPR‐Cas9 biosensing has been developed for DNA detection [131]. The LIG heater fabricated on PI films provides efficient and uniform heating at a constant 65°C with low power consumption, enabling DNA amplification within 30 min.

### 3.5 LIG Bioactive Scaffolds and Transducers for Tissue Engineering and Regenerative Medicine

Tissue engineering and regenerative medicine aim to restore, maintain, or improve tissue function through the synergistic combination of biomaterial scaffolds, cells, and bioactive signals [132]. An ideal scaffold should recapitulate key features of the extracellular matrix (ECM), including appropriate topography, porosity, mechanical compliance, and biochemical cues, while providing a platform for controlled delivery of physical or chemical stimuli that guide cell fate [133]. LIG scaffolds and transducers are emerging as promising bioactive interfaces for these applications owing to their interconnected porous architecture, tunable surface chemistry, electrical conductivity, and compatibility with sensitive cell types, including stem cells.

**FIGURE 8:**
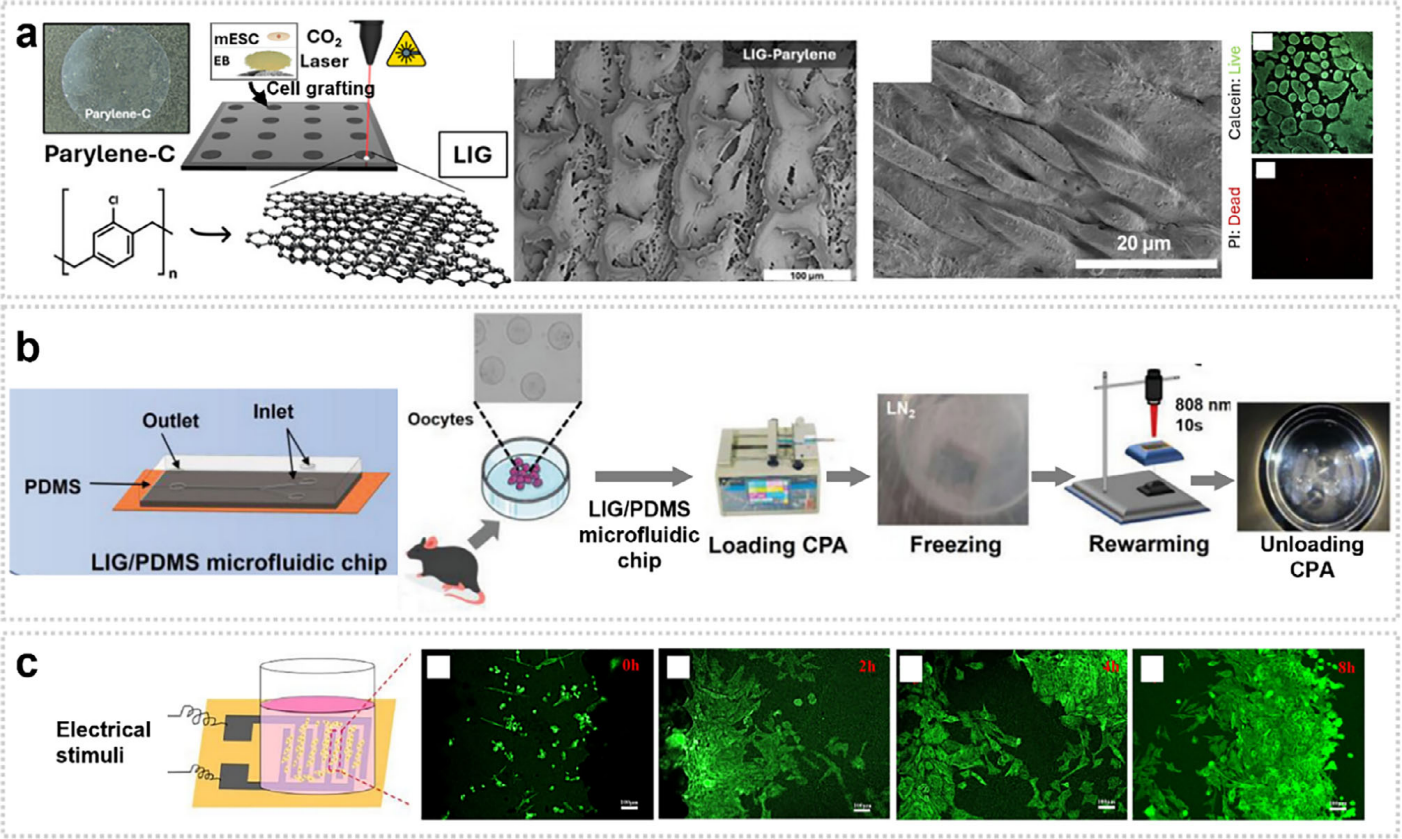
LIG‐based scaffolds for tissue engineering. (a) The anisotropic porous LIG scaffold supports SC culture and differentiation. Reproduced with permission [36]. Copyright 2025, Wiley‐VCH. (b) A LIG/PDMS microfluidic chip with the photothermal effect for oocytes cryopreservation. Reproduced with permission [134]. Copyright 2024, Wiley‐VCH. (c) LIG‐based scaffold and transducer for cell electrostimulation. Reproduced with permission [136]. Copyright 2022, Elsevier.

LIG scaffolds fabricated from Parylene‐C and cellulose paper have been systematically evaluated as culture platforms for mouse embryonic stem cells and embryoid bodies (Figure 8a) [36]. High cell viability exceeding 90% and preserved morphology were observed across multiple mammalian cell types, with successful formation and differentiation of embryoid bodies on LIG scaffold surfaces. The combination of porous and fibrillar microstructure allows LIG scaffolds to mimic the extracellular matrix while providing an electrically addressable interface, suitable for drug screening and cardiac disease modeling.

The tunable surface wettability of LIG scaffolds enables precise control over liquid–solid interactions important for cell‐based fertility applications. A Y‐shaped LIG/PDMS microfluidic chip has been developed for oocyte cryopreservation, where the LIG scaffold provides both tunable hydrophobicity and photothermal conversion (Figure 8b) [134]. By tailoring the channel surface wettability and using 808 nm laser irradiation for rapid rewarming, the chip suppresses ice crystal growth and delays freezing, allowing cryoprotectant concentrations to be reduced by half while achieving oocyte survival rates above 90%.

Neural tissue engineering presents unique challenges due to the electro‐responsive nature of neurons and the limited regenerative capacity of the central nervous system [135]. For active modulation of cell behavior, LIG transducers can be integrated with conductive matrices to deliver electrical stimulation that promotes neural regeneration. LIG interdigitated electrodes coated with PPy form composite scaffolds with enhanced wettability and stable electrochemical performance, which were used for neural tissue engineering applications. PC‐12 neural‐like cells cultured on these LIG/PPy electrodes and subjected to external electric fields of 400 mV cm^−1^ exhibited enhanced proliferation and neuronal differentiation, with average neurite lengths reaching approximately 68 µm after 8 h of stimulation (Figure 8c) [136]. These findings demonstrate the potential of electroactive LIG scaffolds to recapitulate endogenous bioelectric cues essential for neural development and regeneration, offering promising avenues for treating peripheral nerve injuries and neurodegenerative disorders.

### 3.6 Sustainability of LIG‐Based Biomedical Devices

Biomedical devices, including point‐of‐care devices, lab‐on‐a‐chip systems, and single‐use wearable sensors, contact biological samples regularly and require disposable transducers to maintain hygienic operation and analytical reliability. These requirements call for green and sustainable fabrication strategies, and the versatility of LIG substrates highlights its potential as a promising technology to address the agenda.

Various green and sustainable LIG substrates, including lignin, cellulose paper, wood, and cork, have been used to develop biomedical platforms [137]. Lignin‐derived LIG electrodes functionalized by Prussian blue nanomaterials and lactate oxidase have been demonstrated for high‐performance lactate biosensing, establishing a green fabrication route from biomass to functional electrochemical biosensors [53]. Similarly, LIG fabrication on cellulose paper produces nano‐graphitic crystallites that enable fast electron transfer rates for highly efficient electrochemical detection of pH [138]. The use of these green and naturally‐derived substrates for the fabrication of disposable LIG transducers may supports a sustainable lifecycle for the future biomedical device industries [139, 140]. In addition, the LIG fabrication method potentially contributes to the sustainability development of LIG‐based biomedical devices benefit from its ink‐free, mask‐free, and reagent‐free process and required low‐power CO_2_ laser with less energy consumption [141].

## 4 Perspectives

The unique combination of porous architecture, high electrical conductivity, tunable surface chemistry, and facile fabrication of LIG enables multifunctional biomedical integration. As summarized in **Table** **3**, these diverse applications leverage distinct combinations of LIG material properties such as porous architecture for drug loading and bacterial trapping, tunable wettability for microfluidic manipulation, photothermal conversion for controlled release, and antimicrobial, biocompatibility for tissue scaffolding, demonstrating how the intrinsic characteristics of LIG materials can be strategically harnessed to address specific biomedical challenges.

**TABLE 3:** Representative LIG‐based platforms for multifunctional biomedical applications beyond biosensing.

Multifunctional biomedical applications	Materials	Bioactive interface mechanism	Target bioactive elements	Platforms	Performance highlights	Refs.
Anti‐microbial interfaces	LIG/PI	Bactericidal interfaces via membrane stress and oxidative damage through bacterial trapping in high surface area porous architecture (>170 m^2^ g^−1^)	E. coli, S. aureus	N/A	∼93% E. coli and ∼95% S. aureus elimination	[33]
	Nanofibrous LIG/PES	Anti‐adhesion/antibiofilm interface via vertically aligned nanotexture through topographical stress	Bacterial biofilms such as E. coli, S. epidermidis	N/A	Biofilm inhibition >10 days for E. coli and S. epidermidis	[126]
	Quaternary pyridinium‐grafted LIG/PI	Pathogen‐inactivating interface via electrostatic membrane disruption from cationic surface combined with photothermal conversion under ambient light	Broad‐spectrum pathogens such as Escherichia coli, Streptomyces tenebrarius, Candida albicans, HCoV‐OC43 and HCoV‐229E	N/A	Broad‐spectrum pathogen inactivation under ambient light	[127]
	LIG/PI	Electroactive antibiofilm interface via electrical actuation through electrochemical mechanisms	Bacterial biofilms such as Pseudomonas aeruginosa	N/A	Rapid biofilm removal without chemical agents	[128]
	LIG/PI	Photothermal antimicrobial interface via broadband optical absorption enabling light‐triggered localized heating	Airborne pathogens such as E. coli	Face mask	Wireless, self‐reporting antimicrobial protection	[61]
	LIG + PSA + tissue adhesive composite	Electro‐photo‐thermal antimicrobial interface via synergistic Joule heating and light‐induced photothermal conversion	Surface pathogens such as P. aeruginosa	Adhesive tape	Antimicrobial effect without substrate damage	[62]
Smart drug delivery	LIG‐PANI, LIG‐PPy	Stimuli‐responsive therapeutic interface via electrically triggered drug release from conducting polymer matrix	Ciprofloxacin (antibiotic)	Smart bandage	pH sensitivity ∼59 mV/pH; UA range 0–0.9 mm; ciprofloxacin release at 0.6 V	[34]
	LIG/PI + phase‐change material	Thermally‐triggered drug release interface via Joule heating‐induced phase transition enabling controlled drug release and re‐solidification halt	Curcumin	Drug delivery patch	Controllable curcumin dosing; re‐solidification halts release	[129]
	LIG@Fe_3_O_4_	Magnetically‐guided therapeutic interface via magnetic navigation combined with NIR‐triggered photothermal drug release at target sites	Doxorubicin	Magnetic nanorobot	∼50× higher doxorubicin loading vs bare Fe_3_O_4_; ∼96% cargo retention	[68]
Lab‐on‐a‐chip	LIG/PI	Passive biofluidic manipulation interface via tunable wettability enabling capillary‐driven pump‐free fluid manipulation and channel splitting	Ions, pesticides	Microfluidic chip	Multiplexed ion and pesticide detection at picomolar levels	[35]
	LIG/PI + PDMS	Integrated bioanalytical interface with patterned LIG electrodes enabling liposome‐amplified ECL and electrochemical pathogen detection	Cryptosporidium DNA	Lab‐on‐a‐chip	Cryptosporidium DNA LOD: 3 pm (ECL), 47 pm (EC)	[130]
	LIG/cellulose paper	Capillary‐driven bioassay interface via hydrophilic paper matrix with embedded LIG electrodes enabling lateral/vertical flow diagnostics	Alkaline phosphatase, HPV16 DNA	Paper‐based analytical device (µPAD)	ALP detection in serum; HPV16 detection via CRISPR assay	[21]
Nucleic acid amplification	LIG/graphene film + silica glass + PDMS	Thermal cycling biodiagnostic interface via Joule heating enabling rapid thermal cycling (82°C min^−1^) for PCR amplification	Porphyromonas gingivalis DNA (404 bp)	Microfluidic PCR device	Heating rate 82°C min^−1^; 404 bp DNA amplification over 36 cycles	[37]
	LIG/PI + glass	Isothermal amplification interface via uniform resistive heating for LAMP‐based DNA amplification	Target DNA	LAMP‐CRISPR on‐chip system	DNA amplification within 30 min; integrated CRISPR‐Cas9 biosensing	[131]
Tissue engineering and regenerative medicine	LIG/Parylene‐C/cellulose	Cell‐supportive bioactive scaffold via porous fibrillar ECM‐mimicking architecture supporting cell adhesion, culture, and differentiation	Mouse embryonic stem cells, embryoid bodies	Cell culture scaffold	Mouse ESC and embryoid body culture; >90% cell viability	[36]
	LIG/PI + PDMS	Cryoprotective bioactive interface via tunable wettability and laser‐induced photothermal rapid rewarming that suppresses ice crystal formation	Oocytes	Microfluidic cryopreservation chip	Oocyte survival >90% with 50% reduced cryoprotectant	[134]
	LIG/PPy	Electrostimulatory neural interface via conductive PPy‐coated LIG electrodes delivering electrical stimulation (400 mV cm^−1^) for neural differentiation	PC‐12 neural cells	Neural stimulation electrode	PC‐12 neurite extension ∼68 µm after 8 h stimulation	[136]

Abbreviations:

N/A: not applicable; PES: polyether sulfone; PSA: pressure sensitive adhesives; PANI: polyaniline; PPy: polypyrrole; NIR: near‐infrared;

EC: electrochemical; ECL: electrochemiluminescent; ECM: extracellular matrix; PCR: polymerase chain reaction; LAMP: loop‐mediated isothermal amplification; DNA: deoxyribonucleic acid; HCoV: human coronavirus; HPV: human papillomavirus;

CRISPR: clustered regularly interspaced short palindromic repeats; LOD: limit of detection; ALP: alkaline phosphatase; ESC: embryonic stem cells

Building upon these established multifunctional capabilities, several frontiers promise to extend LIG‐based biomedical devices. The integration of optical functionalities could enable future LIG‐based optical and optoelectronic systems [142]. Organ‐on‐a‐chip (OoC) platforms incorporating LIG scaffolds could revolutionize drug screening and disease modeling through electrically active tissue constructs [143, 144]. The emerging concept of 4D LIG introduces time‐dependent and stimuli‐responsive behaviors, pointing toward adaptive devices capable of shape‐morphing, programmable release, and controlled degradation [145, 146]. Finally, the development of advanced artificial intelligence (AI) offers pathways for LIG biomedical applications to enable closed‐loop systems for personalized health monitoring and therapy [147, 148, 149].

### 4.1 Patterned LIG‐Optics

**FIGURE 9:**
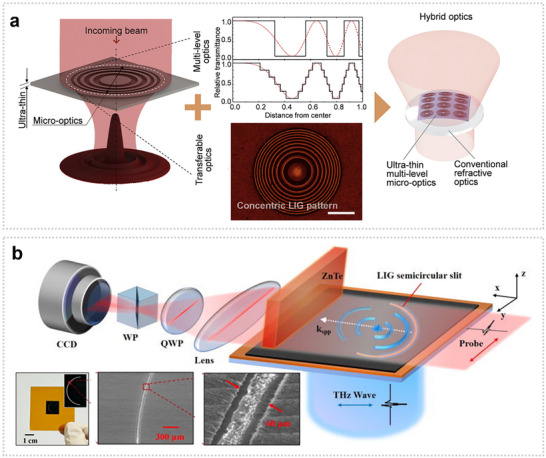
Patterned LIG‐optics. (a) Schematic illustration of concentric transferrable ultra‐thin LIG‐based diffractive micro‐optics array. Reproduced with permission [151]. Copyright 2019, Elsevier. (b) Schematic illustration of porous LIG semicircular slit for Terahertz‐surface plasmon polaritons. Reproduced with permission [152]. Copyright 2022, American Institute of Physics Publishing.

Beyond serving as a conductive electrical transducer, LIG can be patterned to fabricate optical devices, such as diffractive optics, terahertz wave manipulation devices, and dynamic beam steering devices with promising biomedical translations. Through precise femtosecond LIG, the reduction degree of graphene oxide can be tuned at sub‐wavelength scales by modulating laser repetition rate and power, enabling fabrication of Fresnel zone plates with controllable linewidths and transmission profiles as diffractive optical elements (DOEs) [150]. This approach creates multi‐level diffractive lenses surpassing simple binary designs in focusing efficiency. Such lenses can be transferred onto flexible PDMS substrates while maintaining focus performance even on curved surfaces (Figure 9a) [151]. The resulting ultrathin optics (<1 µm thickness) overcomes the bulk and rigidity limitations of conventional refractive elements. In the terahertz regime, LIG's porous architecture enables unique wave manipulation capabilities. The sub‐wavelength porous structure supports terahertz surface plasmon polaritons (THz‐SPPs) with controllable propagation loss (Figure 9b) [152]. Unlike metals, which cannot support SPPs at THz frequencies, LIG provides an effective platform for confining and guiding THz waves. The dynamic optical manipulation through photothermal actuation further expands LIG's optical functionality. Light‐driven diffraction gratings have been demonstrated where LIG acts as a photothermal actuator on PDMS substrates [153]. Upon light irradiation, the LIG layer generates heat that bends the bilayer structure, tuning the diffraction angle in a contactless manner without requiring electrical connections.

### 4.2 LIG Bioactive Scaffolds for Organ‐on‐a‐Chip Platforms

**FIGURE 10:**
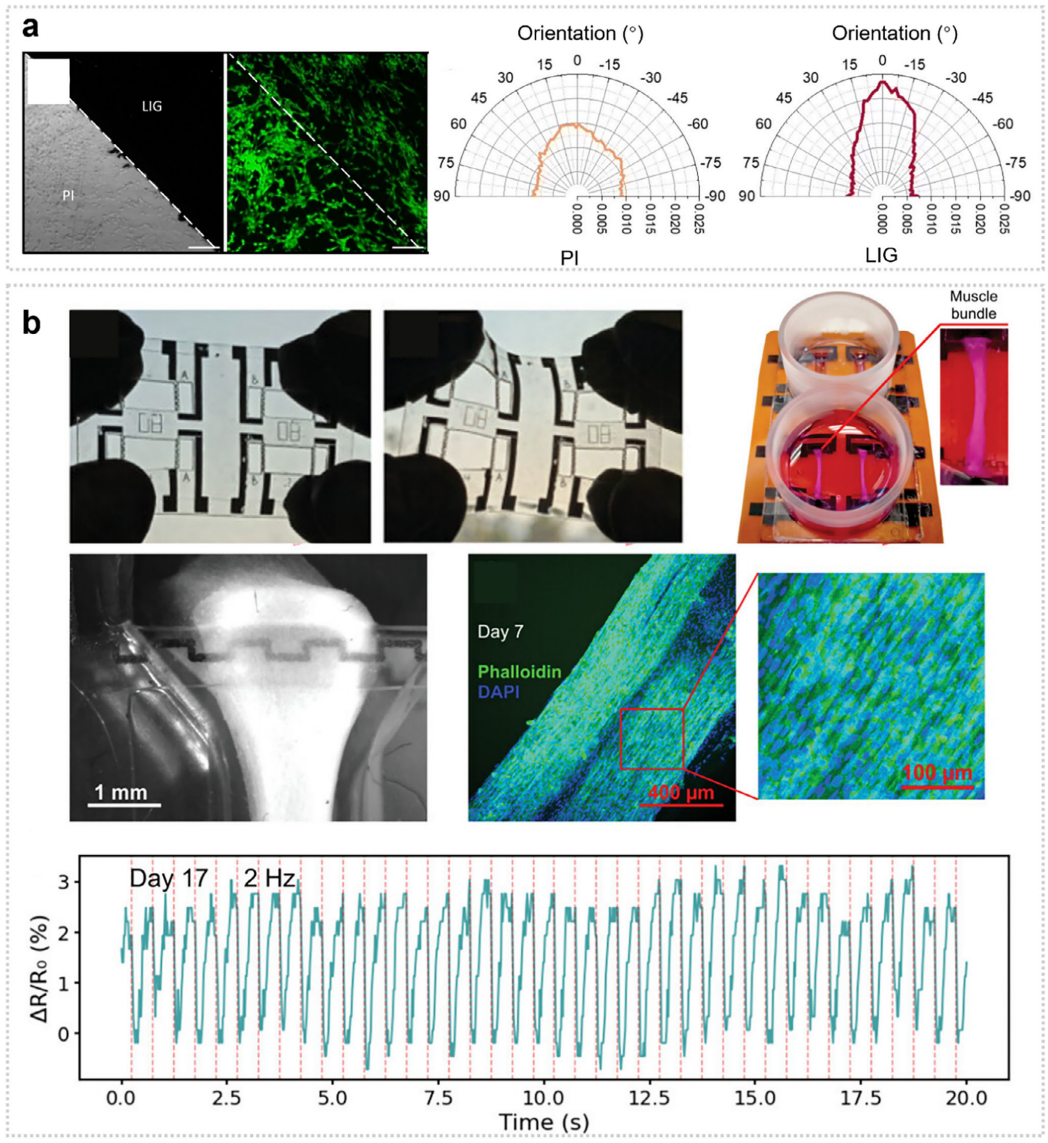
LIG scaffolds and transducers for organ‐on‐a‐chip. (a) LIG transducers with distinct fibril morphology and surface topography that combines bioelectrical functionality with structural support for myogenic differentiation. Reproduced with permission [144]. Copyright 2025, The Royal Society of Chemistry. (b) A muscle‐on‐a‐chip device integrated LIG transducers‐based strain gauges. Reproduced with permission [143]. Copyright 2025, Elsevier.

Organ‐on‐a‐chip (OoC) platforms represent the convergence of bioactive interfaces in regenerative medicine that aim to recapitulate the physicochemical microenvironments of human tissues within miniaturized systems for drug screening, toxicity testing, and disease modeling. As these platforms evolve from static culture models toward dynamic, electrically active systems, there is growing demand for scaffolds that provide structural support with electrical stimulation and real‐time functional readout capabilities [154]. LIG technology enables rapid and benchtop fabrication of micro‐patterned conductive scaffolds that can be directly integrated into OoC devices. The micro‐grooved topography of LIG scaffolds provides contact guidance that directs cellular organization without requiring additional surface modification. This topographic capability, without post‐processing steps like cellular alignment, simplifies OoC fabrication. Unmodified LIG scaffolds have been evaluated for skeletal muscle tissue engineering, where laser‐engraved anisotropic patterns effectively guided alignment of C2C12 myoblasts and promoted formation of highly organized myotubes essential for muscle contractility (Figure 10a) [144]. Furthermore, LIG's compatibility with flexible elastomers enables fabrication of soft, stretchable electronics that mechanically interface with contracting tissues for integrated OoC. A fully integrated muscle‐on‐a‐chip device has been demonstrated where LIG served both as electrodes for electrical stimulation and as PDMS‐embedded strain gauges for the contraction monitoring (Figure 10b) [143]. The LIG‐PDMS strain gauges exhibited stable piezoresistive responses over thousands of cycles, enabling precise quantification of contractile forces generated by cultured muscle bundles under electrical pacing.

In the future, the convergence of LIG‐based structural guidance and integrated sensing will likely drive the development of autonomous OoC platforms that serve as smart bioactive interfaces. By leveraging multidimensional fabrication capabilities, future devices could combine topographically patterned scaffolds for tissue maturation with embedded biosensor arrays for long‐term biochemical and electrophysiological monitoring. Such integrated systems would create regenerative medicine tools capable of continuous assessment of tissue health, drug responses, and disease progression, enabling personalized therapeutic interventions.

### 4.3 4D LIG: Time‐Dependent and Stimuli‐Responsive Bioactive Interfaces

**FIGURE 11:**
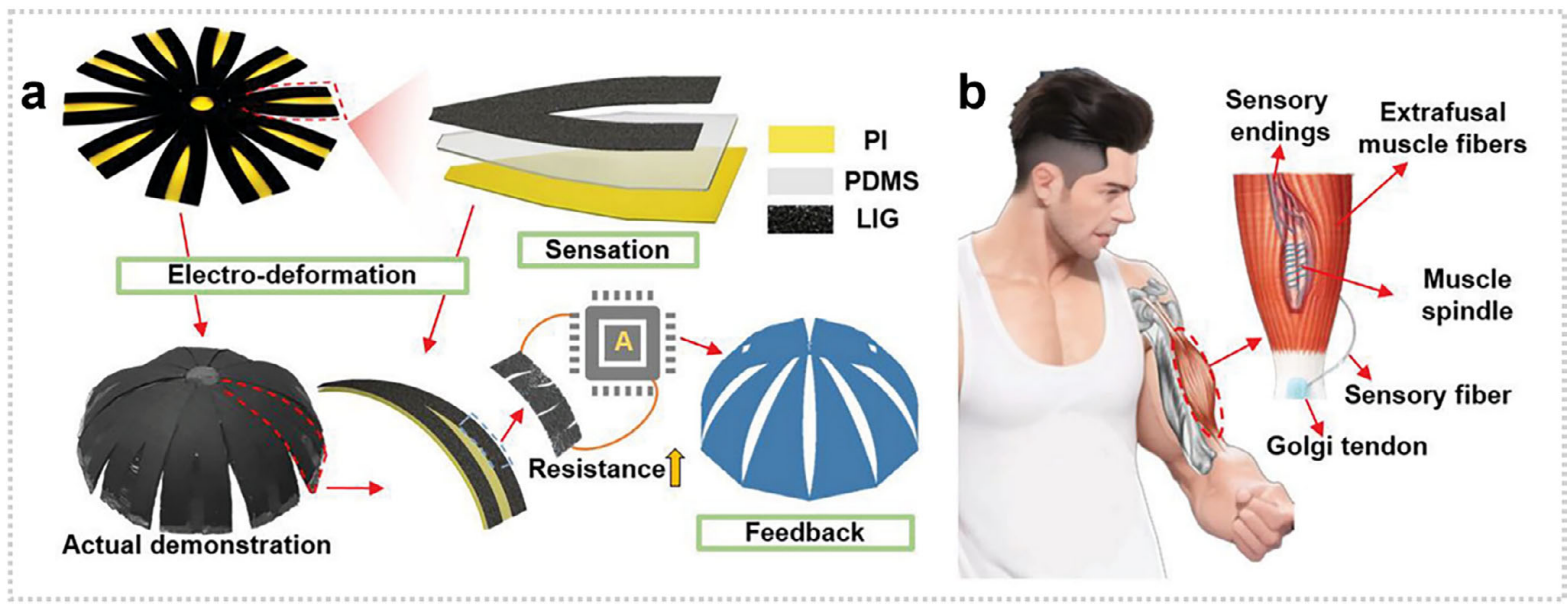
4D LIG transducers‐based actuator. Reproduced with permission [145]. Copyright 2023, Wiley‐VCH.

Besides the 0, 1, 2, and 3D of the LIG, time is introduced as a fourth dimension of LIG. 4D LIG represents an expression of bioactive interfaces acting as a “smart” material that could change shape, property, and function in response to thermal, electrical, or chemical stimuli, that enables shape‐morphing and controlled degradation. Shape‐morphing capabilities allow devices to mechanically adapt to their environment through programmed deformation. LIG transducers‐based electro‐thermal actuators have been developed that simultaneously function as self‐sensing devices where the high conductivity enables efficient conversion of electrical energy into heat for movement, while piezoresistive properties enable the actuators to sense their own deformation (Figure 11) [145]. This dual functionality supports soft robots capable of crawling locomotion and electromagnetic shielding. Furthermore, LIG microheaters integrated with flexible substrates facilitate controlled bending and deformation through electrothermally driven assembly [146]. The rapid, precise control over deformation enabled by LIG's thermal conductivity creates pathways toward bionic actuators responsive to human gestures. In biomedical applications, such shape‐morphing technology could enable smart stents that could self‐expand upon reaching vascular blockages or adaptive tissue scaffolds that mechanically stimulate cell growth through programmed physical actuation, mimicking the natural developmental forces of the body. The 4D concept extends further to the programmed transience, where devices are designed to operate efficiently for a defined period before gradually degrading in vivo. Transient LIG transducers‐based biofuel cells have been developed that harvest biochemical energy from physiological glucose to power implantable electronics before undergoing controlled degradation [59]. These devices demonstrated stable operation for over 28 days with good biocompatibility before gradually breaking down. Such time‐dependent transience is critical for temporary implants, including post‐operative monitors and cardiac pacers, without the need for secondary surgical removal once the patient has recovered.

The 4D LIG establishes a vision for “smart” integrated biosensors and actuators devices that enable closed‐loop theranostic systems that mechanically adapt to tissue interfaces, deliver therapeutics in response to biomarker signals, and safely degrade after completing their mission. This shows the future of regenerative medicine, where intelligent materials seamlessly integrate with biological systems to guide repair, respond to physiological changes, and ultimately transfer functions to regenerated native tissue.

### 4.4 AI‐Powered LIG‐Based Biosensing: Toward Closed‐Loop Personalized Healthcare

The integration of artificial intelligence (AI) with LIG transducers represents a trend from passive data collection to active, intelligent health management. In cardiovascular monitoring, AI models applied to continuous ECG monitoring from LIG electrodes have demonstrated the capacity to predict arrhythmic events with clinically meaningful lead times, enabling pre‐emptive intervention rather than reactive treatment [155]. For infectious disease surveillance, AI models trained on electrochemical signals from LIG‐based biosensors could accelerate outbreak detection by identifying population‐level biomarker trends in near‐real time, complementing the rapid, decentralized testing capability of the LIG point‐of‐care platforms [156]. In cancer diagnostics, AI‐driven multimodal analysis of LIG immunosensor arrays targeting tumor biomarkers such as the CEA, NSE, p53, and SOX2, combined with imaging and clinical metadata, has already demonstrated improved early cancer screening accuracy [106]. In rehabilitation and human‐machine interface applications, AI empowers LIG sensors to decode intricate human motions that are difficult to quantify [155]. Epidermal electrodes combining silver nanowires with LIG transducer patterning have been developed for electromyography (EMG) acquisition, where deep learning models analyzing the multichannel signals achieved 95.4% accuracy in recognizing hand gestures [91]. This AI‐driven pattern recognition offers smart interfaces for analyzing muscle activity, which is important for controlling prosthetics and monitoring rehabilitation progress in personalized physical therapy programs. In postoperative care, a flexible LIG‐based biosensor array integrated with machine learning algorithms has demonstrated 93% accuracy in distinguishing blood from other postoperative drainage fluids, including normal saline, hydrothorax, and ascites [149]. By identifying specific electrochemical signatures and filtering noise, such systems serve as real‐time early warning platforms for hemorrhage risk in thoracic and abdominal surgery patients, significantly reducing false alarms while enabling rapid clinical response.

Thus, the future goal of AI in LIG‐based biomedical devices is to connect diagnostics to therapeutic intervention without human intermediation. We envision future systems where edge computing enables AI to communicate directly on LIG‐based bioactive interfaces, providing ultra‐low latency and privacy‐preserving operation to create intelligent therapeutic systems and revolutionize regenerative medicine. In this landscape, “digital twins” of patients could be created through continuous sensor data analysis, predicting health trajectories and autonomously triggering therapeutic responses when anomalies are detected. Such responses might include activating LIG‐based drug release modules or electrical stimulation patches capable of dynamically adjusting electrical stimulation strategies, drug release profiles, and mechanical stiffness based on real‐time physiological feedback and tissue regeneration progress assessment. This would represent the realization of autonomous, personalized healthcare management, where monitoring, diagnosis, and treatment become a seamless, adaptive continuum tailored to individual patient needs.

## 5 Conclusions

Exploring the dimensional versatility of LIG for multifunctional biomedical applications toward personalized healthcare and regenerative medicine, this review has examined the fundamentals and multidimensional architectures of LIG, and how LIG transducers enable physical, point‐of‐care, portable, wearable, and implantable biosensing. Moving beyond biosensing, we have explored LIG's multifunctional roles in theranostics and bioactive interfaces, including antimicrobial protection, drug delivery, lab‐on‐a‐chip diagnostics, nucleic acid amplification, and tissue engineering, toward personalized healthcare and regenerative medicine. Despite these advances, several important limitations of LIG‐based platforms must be acknowledged. A potential challenge regarding LIG's practicality is its scalability and manufacturability. LIG fabrication is currently performed on benchtop laser systems in laboratory scale with limited throughput [157]. While, we envision the well‐established industrial‐scale laser‐assisted photolithography technique provides an example road map for the future upscale production of LIG‐based biomedical devices [158]. Furthermore, despite the booming research on LIG‐based biomedical devices, their commercialization remains in its early stages. In addition, LIG‐based devices intended for biomedical use must comply with medical device regulations [159]. Looking ahead, addressing these challenges while leveraging the aforementioned strengths of LIG will pave the way for translating laboratory innovations into clinical biomedical platforms.

## Conflicts of Interest

The authors declare no conflicts of interest.

## Data Availability

The data that support the findings of this study are available from the corresponding author upon reasonable request.
